# Using the multidimensional nominal response model to model faking in questionnaire data: The importance of item desirability characteristics

**DOI:** 10.3758/s13428-024-02509-x

**Published:** 2024-09-20

**Authors:** Timo Seitz, Eunike Wetzel, Benjamin E. Hilbig, Thorsten Meiser

**Affiliations:** 1 Department of Psychology, University of Mannheim, L13, 15-17 – room 515, 68161 Mannheim, Germany; 2 University of Kaiserslautern-Landau, Landau, Germany

**Keywords:** Faking, Multidimensional item response theory, Item desirability, Psychological assessment, Psychometrics

## Abstract

Faking in self-report personality questionnaires describes a deliberate response distortion aimed at presenting oneself in an overly favorable manner. Unless the influence of faking on item responses is taken into account, faking can harm multiple psychometric properties of a test. In the present article, we account for faking using an extension of the multidimensional nominal response model (MNRM), which is an item response theory (IRT) model that offers a flexible framework for modeling different kinds of response biases. Particularly, we investigated under which circumstances the MNRM can adequately adjust substantive trait scores and latent correlations for the influence of faking and examined the role of variation in the way item content is related to social desirability (i.e., item desirability characteristics) in facilitating the modeling of faking and counteracting its detrimental effects. Using a simulation, we found that the inclusion of a faking dimension in the model can overall improve the recovery of substantive trait person parameters and latent correlations between substantive traits, especially when the impact of faking in the data is high. Item desirability characteristics moderated the effect of modeling faking and were themselves associated with different levels of parameter recovery. In an empirical demonstration with *N* = 1070 test-takers, we also showed that the faking modeling approach in combination with different item desirability characteristics can prove successful in empirical questionnaire data. We end the article with a discussion of implications for psychological assessment.

**Supplementary Information:**

The online version contains supplementary material available at 10.3758/s13428-024-02509-x.

When filling out a self-report personality questionnaire, test-takers have the opportunity to give overly positive self-descriptions (Paulhus, 2002). Especially when the questionnaire is part of an assessment whose results have important consequences for test-takers, a substantial proportion of test-takers can be expected to engage in faking, that is, to deliberately distort responses according to social desirability (e.g., Griffith & Converse, 2011; König et al., 2011). Unless the effect of faking is accounted for, faking can harm various psychometric properties of a test (Ziegler et al., 2011). Also, when it comes to personality assessments in actual high-stakes situations, faking can play a decisive role in decisions about hiring and promotion (e.g., Mueller-Hanson et al., 2003).

In this article, we address the response bias of faking by means of item response theory (IRT) modeling. In particular, we examine under which circumstances the multidimensional nominal response model (MNRM; Takane & de Leeuw, 1987; see Falk & Cai, 2016; Seitz et al., 2023), which offers a framework for flexibly modeling different kinds of response biases, can adequately adjust substantive trait scores and latent correlations between substantive traits for the influence of faking. We hereby focus on the role of variation in the way item content is related to social desirability (i.e., item desirability characteristics) and investigate how such variation can facilitate the modeling of faking and counteract its adverse effects.

## Background: Faking in personality assessment

Faking is also known as impression management and represents the deliberate form of socially desirable responding (SDR) in Paulhus’ (1984) well-known two-component model of SDR. Research has repeatedly shown that faking can have numerous effects on a test’s psychometric properties (Ziegler et al., 2011). For instance, depending on whether desirable (undesirable) traits are measured, faking leads to considerably inflated (deflated) item and scale scores (e.g., Birkeland et al., 2006; Viswesvaran & Ones, 1999). A shift in item and scale scores would not be problematic for the assessment of interindividual differences if the range of possible scores was unlimited and if all test-takers shifted their scores by an equal amount. However, because self-report questionnaires often use a Likert-type rating scale with a limited number of response categories, inflated (deflated) scores are typically associated with heavily skewed score distributions and ceiling (floor) effects. Also, many studies have pointed out that test-takers differ in their propensity to fake (see Griffith & Converse, 2011). This implies that test-takers shift their scores by an unequal amount. For instance, using a randomized response technique, König et al. (2011) estimated that 32% of job applicants in the U.S. exaggerate their positive attributes in application settings whereas others do not. Likewise, when retesting job applicants under anonymous conditions (i.e., in a low-stakes context), Griffith et al. (2007) found that 30-50% of applicants had significantly elevated their scores in the preceding application (i.e., in a high-stakes context; see also Donovan et al., 2003). Such interindividual differences between test-takers imply rank-order changes and eventually alter selection decisions based on test scores (e.g., Mueller-Hanson et al., 2003). These rank-order changes can in turn have different consequences for a test’s criterion-related validity, depending on how the degree of faking is correlated with the criterion variable of interest (see Komar et al., 2008). Moreover, interindividual differences in faking constitute an additional source of variance in item responses, leading to inflated intercorrelations between scales that measure desirable traits (e.g., Ellingson et al., 1999; Klehe et al., 2012; Schmit & Ryan, 1993). Faking can hence diminish construct validity in terms of a distorted discriminant validity between scales, which makes nuanced profiles of scores in a personality inventory unlikely.

Over the past decades, faking and SDR have been extensively studied by psychologists and survey methodologists. A prominent approach has been to measure SDR through designated SDR scales (see Paulhus, 2002, for an overview). These scales contain items that capture desirable behaviors hardly shown by anyone as well as undesirable behaviors that are in fact very common. Endorsing many of the former and few of the latter items would yield a high score on an SDR scale. In high-stakes assessments, SDR scales of impression management as well as related measures have been widely used to quantify faking and correct substantive trait scores for the assumed bias (Goffin & Christiansen, 2003). However, many studies have demonstrated that SDR scales are confounded with substantive trait variance and hence measure, at least to a certain degree, true personality attributes as opposed to only response bias, which makes it inappropriate to partial SDR scale scores from personality scale scores in order to achieve “pure” measures of personality (e.g., de Vries et al., 2014; McCrae & Costa, 1983; Müller & Moshagen, 2019).

Along with SDR scales and other so-called validity scales, several indirect measures have been developed to detect faking (see Goldammer et al., 2023). These include measures of response inconsistency such as person-fit indices in IRT models (e.g., LaHuis & Copeland, 2009), exploratory mixture models to identify latent faking classes (e.g., Zickar et al., 2004), and measures of extreme responding (e.g., Sun et al., 2022). However, these measures focus on the detection of faking and primarily yield an additional piece of information regarding individual test-takers. It also remains questionable how well these measures are suited to adequately adjust substantive trait scores for faking. Hence, it is appealing to have a latent variable model that directly incorporates information on the degree of faking in the estimation of model parameters and test-takers’ substantive trait levels.

## The multidimensional nominal response model to account for faking

To model nominal (i.e., categorial) item responses, Bock (1972) proposed an IRT model in which item responses are assumed to be influenced by a single latent dimension representing the trait of interest. Takane and de Leeuw (1987) extended this model for the case of multiple latent dimensions affecting item responses. In this multidimensional generalization, the probability of test-taker *n* choosing response category *k* out of a set of *K*+1 categories on item *i* is modeled with the following multinomial logistic function:

1 $${p}\mathrm({Y}_{ni} = {k} \vert{{\varvec{\theta}}}_{n}, {{\varvec{\gamma}}}_{i}, {\varvec{\alpha}}_{i}, {\varvec{S}}_{i}) = \frac{\mathrm{exp}((\varvec{\alpha}_{i}\circ{\varvec{s}}_{ik})^{\prime}{{\varvec{\theta}}}_{n} + {\gamma}_{ik})}{\sum\nolimits_{{m}= \mathrm{0} }^{K}\mathrm {exp}((\varvec{\alpha}_{i}\circ{\varvec{s}}_{im})^{\prime}{{\varvec{\theta}}}_{n} + {\gamma}_{im})}$$

with $${{\varvec{\theta}}}_{n}=\left(\begin{array}{c}{\theta }_{n1}\\ \vdots\\ {\theta }_{nd}\\ \vdots \\ {\theta }_{nD}\end{array}\right)$$, $$\varvec{\gamma}_{i} = (\begin{array}{c}{\gamma}_{i0}\ \ \ \cdots \ \ \ {\gamma}_{ik}\ \ \ \cdots \ \ \ {\gamma}_{iK}\end{array})$$, $$\varvec{\alpha}_{i} = \left(\begin{array}{c}{\alpha}_{i1}\\ \vdots\\ {\alpha}_{id}\\ \vdots \\ {\alpha}_{iD}\end{array}\right)$$, and $$\varvec{S}_i=\begin{pmatrix}s_{i10}&\dots&s_{i1k}&\dots&s_{i1K}\\\vdots&&\vdots&&\vdots\\s_{id0}&\dots&s_{idk}&\dots&s_{idK}\\\vdots&&\vdots&&\vdots\\s_{iD0}&\dots&s_{iDk}&\dots&s_{iDK}\end{pmatrix}$$. 

*Y*_*ni*_ is a discrete random variable that reflects the response of test-taker *n* on item *i* (*Y*_*ni*_ ∈ {0, 1, …, *k*, …, *K*}), *k* denotes the realization of *Y*_*ni*_, ***θ***_*n*_ is a *D*-dimensional column vector of test-taker *n*’s levels on the *D* dimensions, and ***γ***_*i*_ is a (*K*+1)-dimensional row vector of item- and category-specific intercepts. This parametrization of the MNRM (Falk & Cai, 2016; Thissen & Cai, 2016) also includes item-specific slopes *α*_*id*_ (collected in the *D*-dimensional column vector ***α***_*i*_) representing the relation between item *i* and dimension *d* as well as item- and category-specific scoring weights *s*_*idk*_ (collected in the *D*×(*K*+1)-dimensional matrix ***S***_*i*_) representing the relation between dimension *d* and category *k* at item *i*. The symbol ◦ denotes the Hadamard product which links ***α***_*i*_ and ***s***_*ik*_ (a column vector in matrix ***S***_*i*_). That is, parameters pertaining to the same dimension *d* are multiplied before the resulting column vector is transposed and multiplied by ***θ***_*n*_. This leads to a sum of products α_*id*_ *s*_*idk*_ *θ*_*nd*_ over the *D* dimensions. After *γ*_*ik*_ is added to this sum, the resulting term is divided by the sum of these terms for the *K*+1 categories to yield the probability of an item response. Hence, the MNRM falls into the class of divide-by-total IRT models (Thissen & Steinberg, 1986). For model estimation, identification constraints must be imposed (see Falk & Cai, 2016, for details). The *D* latent dimensions are typically assumed to be multivariate normally distributed with expectation vector ***µ*** = **0** and variance-covariance matrix ***Σ*** in which all variances are fixed to 1. The intercept of the first category is usually fixed to 0 for all items.

If one has theoretical assumptions on relations between dimensions and categories, one can also specify scoring weights a priori. For latent dimensions representing substantive traits, scoring weights of items measuring the respective substantive trait are usually set to equally spaced values (e.g., $$\left(\begin{array}{ccccccc}{0}& {1}& {2}& {3}& {4}& {5}& {6}\end{array}\right)$$ in the case of a seven-point Likert scale), reflecting the assumption that higher response categories are triggered by higher substantive trait levels. Such a model is essentially a partial credit model (PCM; Masters, 1982) or a generalized partial credit model (GPCM; Muraki, 1992), depending on whether between-item equality constraints are imposed on slope parameters. Along with latent dimensions representing substantive traits, response bias dimensions can be specified. Multiple studies have used the MNRM to model response styles along with substantive traits (e.g., Bolt & Newton, 2011; Wetzel & Carstensen, 2017; see Henninger & Meiser, 2020, for an overview). Response styles are tendencies of test-takers to prefer certain response categories irrespective of item content (see Van Vaerenbergh & Thomas, 2013, for an overview). One prominent example is extreme response style (ERS), which reflects the tendency to prefer the highest or lowest category of a rating scale. Based on the definition of a particular response style, one can specify scoring weights of the respective response style dimension. For instance, in the case of a seven-point Likert scale, the scoring weight vector $$\left(\begin{array}{ccccccc}{1}& {0}& {0}& {0}& {0}& {0}& {1}\end{array}\right)$$ can be specified for ERS, reflecting the assumption that extreme rating scale categories are triggered by high ERS levels. Response styles are by definition independent of item content. Hence, the same scoring vector is usually specified for every item of the test.

To additionally account for the response bias of faking, one can add another latent dimension to the model. Because scoring weights code the relation between a dimension and a category on a particular item, scoring weights of the faking dimension can be set to values that reflect the desirability levels of response categories on a given item (Falk & Cai, 2016; see Seitz et al., 2023). As Kuncel and Tellegen (2009) demonstrated, the pattern of the relationship between response categories and social desirability differs between personality items. Hence, in contrast to substantive trait and response style dimensions, scoring weights of the faking dimension have to be specified in an item-specific way. Such a model explicitly accounts for the possibility that desirability does not increase or decrease monotonically with response categories for some items. Thus, items at which moderate levels of agreement are most desirable can be modeled, which constitutes an important extension over other recent faking models (e.g., Böckenholt, 2014; Brown & Böckenholt, 2022; Hendy et al., 2021; Leng et al., 2020; Ziegler & Bühner, 2009).

Like other psychometric models that account for response tendencies of test-takers, the presented faking model treats faking as a normally distributed latent variable. Since latent variables do not have a natural origin and scaling, the latent mean as well as the latent variance of all dimensions need to be defined for model identification. In this article, we set the latent mean to 0 and the latent variance to 1 for all dimensions. Test-takers’ scores can thus be interpreted in terms of *z*-scores and, similar to regression analyses, intercepts represent propensities toward response categories for test-takers with mean scores on all latent dimensions. Since the fixations for model identification are arbitrary, fixing the latent faking mean to 0 does not imply that a positive versus negative faking score reflects socially desirable (“faking good”) versus socially *un*desirable responding (“faking bad”). It rather reflects that a test-taker’s faking degree is above versus below average in the analyzed dataset.^1^ In the same vein, a faking score of 0 does not imply the absence of faking but a faking degree that corresponds to the average extent of faking in the respective sample.

Applying the presented model to a sample of bank apprentice applicants taking a Big Five personality test as part of their application, Seitz et al. (2023) provided evidence for the utility of the MNRM to model faking in a high-stakes assessment. To get scoring weights for the faking dimension, the authors had asked pilot study participants to rate each response category of each item of the personality test regarding desirability in the context of an apprenticeship in the financial industry. The model including a faking dimension with scoring weights collected in the pilot study fit the data significantly better than a model only accounting for substantive traits and response styles and improved the discriminant validity of the substantive trait scales by disinflating latent correlations. Also, comparing job applicants and job incumbents, the authors found initial evidence that the model can capture the assumed influence of faking and adjust person parameters of substantive traits in the expected direction.

### Open questions

Since the study by Seitz et al. (2023) was focused on an empirical application of the model to a single high-stakes dataset and featured only a quasi-experimental validation, essential psychometric properties of the faking modeling approach are still unknown. For instance, Seitz et al. (2023) only demonstrated that the model can adjust substantive trait person parameters in the expected direction. It remained unclear if the adjustments in fact lead to more accurate estimates of the true person parameters. To answer this question, it is necessary to know the underlying population model, which is the case in simulation studies but not in applications to empirical data. Also, Seitz et al.’s (2023) empirical application mainly showed that the faking modeling approach can bring inflated latent correlations between substantive traits closer to 0. Whether it really affords more precise representations of intercorrelations between substantive traits, however, requires further research.

Along with these questions regarding the general superiority of the faking modeling approach, facilitating and limiting factors of the model’s superiority have yet to be examined. For example, considering that faking is specified by setting scoring weights to desirability levels of response categories, desirability characteristics of the items used to model faking can be assumed to play a crucial role. Even though Kuncel and Tellegen (2009) found that items of regular personality tests do differ in terms of the relationship between response categories and desirability, the usual case is that higher categories are associated with higher desirability levels.^2^ For instance, for 87.5% of the items in Seitz et al. (2023) and 94.5% of the items in Kuncel and Tellegen (2009), the trajectory of the relation between categories and desirability had a significantly positive linear trend. That is, personality items are in most cases constructed in a way that descriptive aspects of the trait of interest (i.e., substantive trait levels) coincide with evaluative aspects (i.e., desirability levels; Peabody, 1967). This implies that high scores can be due to a high substantive trait level, a high tendency to respond according to desirability (i.e., faking), or both, unless test-takers’ faking tendency is statistically accounted for. Transferred to modeling faking by means of the MNRM, however, a situation with confounded descriptive and evaluative aspects causes high collinearity between the scoring weight vectors of the substantive trait and faking dimensions. One can assume that substantive traits and faking become increasingly hard to disentangle the more items there are with highly overlapping scoring weight vectors. In the extreme case, namely, if only one substantive trait was modeled and all items exhibited perfectly linear desirability trajectories in the direction of the substantive trait, the model would even be not identified. If descriptive and evaluative aspects were in turn not associated across items, scoring weight vectors of substantive traits and faking would not show collinearity, which arguably facilitates the modeling. Also, considering that a high faking tendency would in this case not lead to high responses on every item, high scores on a scale would be a better indication of high substantive trait levels even if faking was not accounted for. Hence, item desirability characteristics can be expected to moderate the effect of modeling faking with the MNRM.

The present research addresses the open questions regarding the MNRM approach to modeling faking by means of a simulation and an empirical study. In the simulation, it is examined if and under which conditions modeling faking along with substantive traits and response styles effectively outperforms a model without a faking dimension in terms of a) the recovery of substantive trait person parameters and b) the recovery of latent correlations between substantive traits. The empirical part in turn investigates whether the faking modeling approach in combination with different item desirability characteristics also proves successful in empirical questionnaire data.

## Simulation study

### Simulation design

**Fig. 1:**
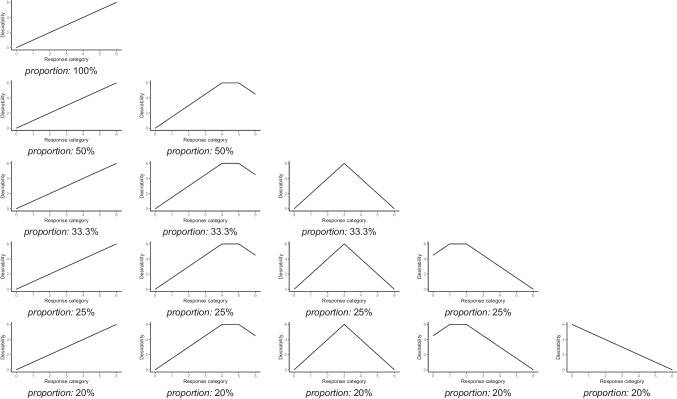
Compositions of desirability trajectories *. *The proportions of desirability trajectories refer to the proportions within each substantive trait scale. The depicted desirability trajectories implied the following scoring weight vectors of faking: $$\left(\begin{array}{ccccccc}{0}& {1}& {2}& {3}& {4}& {5}& {6}\end{array}\right)$$ for monotonically increasing trajectories; $$\left(\begin{array}{ccccccc}{0}& \mathrm{1.5}& {3}& \mathrm{4.5}& {6}& {6}& \mathrm{4.5}\end{array}\right)$$ for nonmonotonically increasing trajectories; $$\left(\begin{array}{ccccccc}{0}& {2}& {4}& {6}& {4}& {2}& {0}\end{array}\right)$$ for inverted-U-shaped trajectories; $$\left(\begin{array}{ccccccc}\mathrm{4.5}& {6}& {6}& \mathrm{4.5}& {3}& \mathrm{1.5}& {0}\end{array}\right)$$ for nonmonotonically decreasing trajectories; $$\left(\begin{array}{ccccccc}{6}& {5}& {4}& {3}& {2}& {1}& {0}\end{array}\right)$$ for monotonically decreasing trajectories. In conditions in which the proportions implied non-integer numbers of items, we rounded the respective proportions up or down to the next integer such that a symmetrical distribution of desirability trajectories was ensured

In the present simulation, we manipulated several factors to simulate different conditions with respect to item and test construction aspects, sample size, as well as the presence of response styles and the impact of faking in the data. Irrespective of the condition, we generated data in which five substantive traits were measured by different sets of items on a seven-point Likert scale. To examine the effects of different item desirability characteristics, we compared five item compositions characterized by different levels of variety of desirability trajectories (see Fig. 1): In the first composition, all items within a substantive trait scale had a monotonically increasing desirability trajectory (i.e., highest desirability for the highest category). In the second composition, half of the items within a substantive trait scale had a desirability trajectory as in the first composition, whereas the other half had a nonmonotonically increasing desirability trajectory (i.e., desirability generally increased with higher categories but peaked at the non-extreme agreement categories and then decayed). In the third composition, two-thirds of the items within a substantive trait scale had desirability trajectories as in the second composition, whereas one-third had an inverted-U-shaped desirability trajectory (i.e., the midpoint category of the rating scale had highest desirability). In the fourth composition, three-quarters of the items within a substantive trait scale had desirability trajectories as in the third composition, whereas one-quarter had a nonmonotonically decreasing desirability trajectory (i.e., lower categories were generally associated with higher desirability, but with a peak at the non-extreme disagreement categories and a decay at the extreme disagreement category). In the fifth composition, four-fifths of the items within a substantive trait scale had desirability trajectories as in the fourth composition, whereas one-fifth had a monotonically decreasing desirability trajectory (i.e., highest desirability for the lowest category). Note that the different desirability trajectories only determined how faking manifested in item responses. Concerning substantive traits, higher response categories were always associated with higher substantive trait levels for all items, such that the five item compositions represented different levels of collinearity between scoring weight vectors of substantive traits and faking.

Along with item desirability characteristics, we varied the number of items per substantive trait scale (6 vs. 12) and the number of simulated test-takers (500 vs. 1000 vs. 2000). Also, we manipulated the presence of response styles (no response styles vs. ERS) and the impact of faking in the data. Considering the faking impact, we varied the extent to which the faking dimension manifested in item responses (no manifestation vs. low manifestation vs. high manifestation) to examine how this affects parameter recovery in different models.

### Data generation and fitted models

To generate the data for the respective simulation conditions, we proceeded as follows (the entire simulation syntax can be found at https://osf.io/ms57p/):

1. Item-specific slopes *α*_*id*_: Slopes of substantive trait dimensions were drawn from *U*(min = 0.25, max = 0.75). In conditions in which ERS was present, slopes of the ERS dimension were drawn from *N*(*µ* = 0.25, *σ* = 0.1), reflecting values of a typical behavior of response styles (cf. Falk & Cai, 2016). In conditions without ERS, ERS slopes were set to 0. Regarding the impact of faking, slopes of the faking dimension were set to 0 in conditions with no faking impact, whereas faking slopes were drawn from *U*(min = 0, max = 0.5) in low-faking impact conditions and from *U*(min = 0.25, max = 0.75) in high-faking impact conditions. That is, faking slopes were specified to be on average as high as substantive trait slopes in conditions with a high faking impact and on average half as high as substantive trait slopes in conditions with a low faking impact.
2. Scoring weights *s*_*idk*_: Scoring weights of substantive traits and ERS were set to values as described in the introduction of the MNRM, whereas scoring weights of ERS were linearly transformed to a range from 0 to 6 to ensure a common metric of scoring weights across dimensions (cf. Falk & Ju, 2020). Scoring weights of faking depended on the respective condition of item desirability characteristics, that is, on the composition of desirability trajectories. The respective scoring weight vectors of faking can be found in Fig. 1.
3. Item-/category-specific intercepts *γ*_*ik*_: The intercept of the first category was fixed to 0 for all items. The remaining intercepts were generated by sampling item- and category-specific threshold values *τ*_*ik*_ from *MVN*(***µ*** = $\overline{{{\tau}}}$, ***Σ*** = ***T***), where $$\overline{{\varvec{\tau}}} =(\begin{array}{c}{-1.5}\ \ \ \ \ {- 0.9}\ \ \ \ \ {-0.3}\ \ \ \ \ {0.3}\ \ \ \ \ {0.9}\ \ \ \ \ {1.5}\end{array})^{\prime}$$ and $${\varvec{T}} = {\mathrm{diag}}(\begin{array}{c}{0.7}\ \ \ \ \ {0.7}\ \ \ \ \ {0.7}\ \ \ \ \ {0.7}\ \ \ \ \ {0.7}\ \ \ \ \ {0.7}\end{array})$$, and transforming them to cumulative thresholds that reflect intercepts: ${\gamma}_{ik}= -\sum\nolimits_{{m}= 0 }^{k}\tau_{im}$. These population values were chosen to generate item response distributions that could cover all response categories in the present constellation.
4. Person parameters *θ*_*nd*_: Person parameters with a sample size depending on the respective condition were drawn from *MVN*(***µ***, ***Σ***), where $${\mu}= \left(\begin{array}{ccccccc}{0}& {0}& {0}& {0}& {0}& {0}& {0}\end{array}\right)$$ and ***Σ*** was the variance-covariance matrix from Table 1. Latent variances were fixed to 1 for all dimensions. Latent covariances between substantive traits were set to values from van der Linden et al.’s (2010) meta-analysis on intercorrelations between the Big Five personality factors. ERS was set orthogonal to all substantive traits and faking. Latent covariances between faking and the five substantive traits were set to .00, .10, –.10, .30, and –.30.
5. Using the generated item and person parameters, item responses were simulated based on the multinomial logistic function in Eq. (1).
6. Steps 1 to 5 were replicated such that 100 datasets were generated per condition.

**Table 1:** Latent correlations between substantive traits, ERS, and faking used for data generation in the simulation

	θ_1_	θ_2_	θ_3_	θ_4_	θ_5_	θ_ERS_	θ_Faking_
θ_1_	1						
θ_2_	*.26*	1					
θ_3_	*.29*	*.43*	1				
θ_4_	*.36*	*.36*	*.43*	1			
θ_5_	*.43*	*.21*	*.20*	*.17*	1		
θ_ERS_	.00	.00	.00	.00	.00	1	
θ_Faking_	.00	.10	–.10	.30	–.30	.00	1

*Note.* Latent correlations between substantive traits θ_1_ to θ_5_ are values from van der Linden et al.’s (2010) meta-analysis on intercorrelations between the Big Five (Neuroticism coded as Emotional Stability). The assignment of these correlations (printed in italics) to the ten substantive trait pairs was randomized between replications. ERS = extreme response style

All steps were carried out in the *R* environment (version 4.2.3) using the packages *MASS* (Venables & Ripley, 2002), *mirt* (Chalmers, 2012), and *SimDesign* (Chalmers & Adkins, 2020). Since research has repeatedly demonstrated the importance and stability of response styles like ERS in different assessment contexts (e.g., Bolt & Newton, 2011; LaHuis et al., 2019; Wetzel & Carstensen, 2017; Wetzel et al., 2016), a model accounting for substantive traits, ERS, and faking was compared to a model only accounting for substantive traits and ERS. These two models of interest were fitted to all 100 simulated datasets per condition.^3^ For model identification, the above-described constraints were imposed. Scoring weights of the substantive trait and ERS dimensions were specified as described above. To emulate that scoring weights of the faking dimension are usually unknown in non-simulated item sets and can hence only be approximated (e.g., by pilot study ratings), we contaminated faking scoring weights used for model estimation with random noise.^4^ Because of high dimensionality, models were estimated using the Metropolis-Hastings Robbins-Monro (MH-RM) algorithm (Cai, 2010) as implemented in the *mirt* package. The MH-RM algorithm constitutes an estimation procedure that features elements from Markov chain Monte Carlo (MCMC) techniques and stochastic approximation methods and thereby converges to the maximum likelihood solution. To estimate person parameters in the high-dimensional models, maximum a-posteriori (MAP) scores were computed (see Thissen & Wainer, 2001).

### Analysis

As outlined above, the simulation study should assess the performance of the faking modeling approach compared to a model not accounting for faking in recovering substantive trait person parameters and latent correlations between substantive traits under different circumstances. As the complete simulation design comprised 5 (*Item Desirability Characteristics*) × 2 (*Test Length*) × 3 (*Sample Size*) × 2 (*Presence of Response Styles*) × 3 (*Faking Impact*) = 180 conditions, we calculated effect size estimates in an analysis of variance (ANOVA) framework with the respective recovery statistic as dependent variable and the five simulation factors as well as the respective model as independent variables to evaluate the contribution of each factor and potential interactions. Since the two models of interest were fitted to the same data within a replication, we treated the factor *Model* as a repeated-measures factor. To quantify proportions of variance explained in this multifactorial mixed ANOVA, we used the R package *afex* (Singmann et al., 2023) to compute the generalized *η*^2^ statistic (${\eta}_{G}^{2}$) that provides effect size estimates that are comparable across various research designs (Olejnik & Algina, 2003). As there are no established conventions for interpreting ${\eta }_{G}^{2}$ effect size estimates, we interpreted ${\eta }_{G}^{2}$ values of main effects and interactions within a given ANOVA in a relative manner. Considering the large effect sizes of some main effects and interactions, we regarded ${\eta }_{G}^{2}$ values smaller than .05 as negligible.

Concerning the recovery of substantive trait person parameters, we considered the correlation between estimated and true person parameters. In particular, the Fisher-*z*-transformed Pearson correlation between the estimated and true person parameters was computed for all five substantive traits within each replication of every condition to convert correlation coefficients into an asymptotic normal distribution. For the recovery of latent correlations, we looked at bias as well as root mean square error (RMSE). For bias, the deviation between estimated and true latent correlations was calculated for the ten substantive trait pairs *j* and then averaged within each replication of every condition:

2 $${\mathrm{bias}}_{rep} = \frac{1}{{10}}\sum\limits_{j}{{(\widehat\rho}_{j} - {\rho}_{j})}.$$

For RMSE, the deviation was squared and averaged across the ten substantive trait pairs before the square root was taken, which served as an indicator of estimation precision within each replication of every condition:

3 $${\mathrm{RMSE}}_{rep}= \sqrt{\frac{1}{{10}}\sum\limits_{j}{\left({\widehat\rho}_{j}-{\rho}_{j}\right)}^{2}} .$$

### Simulation results

#### Recovery of substantive trait person parameters

Using the above-described ANOVA framework to analyze the Fisher-*z*-transformed correlations between estimated and true person parameters of substantive traits, we found that *Model* had a main effect of ${\eta}_{G}^{2}$ = .437, indicating considerable differences in person parameter recovery between the model ignoring faking and the model accounting for faking across conditions. The main effects of *Item Desirability Characteristics* (${\eta}_{G}^{2}$ = .565), *Test Length* (${\eta}_{G}^{2}$ = .678), and *Faking Impact* (${\eta}_{G}^{2}$ = .864) were also meaningful. In contrast, the main effects of *Sample Size* (${\eta}_{G}^{2}$ = .003) and *Presence of Response Styles* (${\eta}_{G}^{2}$ = .006) as well as all interactions including at least one of these two factors (${\eta}_{G}^{2}$ s < .005) were negligible. *Test Length* also did not meaningfully interact with the other factors (${\eta}_{G}^{2}$ s < .040), except for a two-way interaction with *Faking Impact* (${\eta}_{G}^{2}$ = .132). Regarding the factors *Model*, *Item Desirability Characteristics*, and *Faking Impact*, there was a pronounced three-way interaction (${\eta}_{G}^{2}$ = .204) that qualified the three two-way interactions between these three factors (.139 < ${\eta}_{G}^{2}$ s < .434; effect size estimates of all main effects and interactions can be found in Table S.I.1 in Supplement I).^5^

**Fig. 2:**
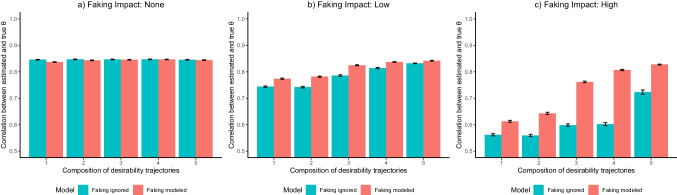
Simulation study: Recovery of substantive trait person parameters*. *The depicted recovery of substantive trait person parameters is for the representative case of six items per substantive trait scale, a sample size of 1000, and extreme response style (ERS) being present in the data. Models ignoring faking only included dimensions for substantive traits and ERS, whereas models accounting for faking also included a faking dimension. Results are aggregated across the five substantive traits used in the simulation. Values reflect the back-transformed mean of the Fisher-*z*-transformed correlations between estimated and true person parameters across replications within a condition. Error bars represent the standard error of the mean

Considering that higher-order interactions with *Test Length*, *Sample Size*, and *Presence of Response Styles* were negligible, Fig. 2 depicts the three-way interaction between *Model*, *Item Desirability Characteristics*, and *Faking Impact* for the representative case of 6 items per substantive trait scale, a sample size of 1000, and ERS being present in the data: When there was no faking in the data (see Fig. 2a), the model ignoring faking and the model accounting for faking did not differ regarding person parameter recovery, irrespective of item desirability characteristics. When the faking impact was low (see Fig. 2b), the model accounting for faking recovered person parameters better than the model ignoring faking. However, effects were rather small and almost vanished when item sets were composed of all desirability trajectory types. When the faking impact was high (see Fig. 2c), differences between the models were more pronounced, such that the model accounting for faking performed considerably better than the model ignoring faking in all compositions of desirability trajectories. Note also the main effect due to the different item desirability characteristics: Unless faking was absent in the data, person parameter recovery improved in item compositions with more variety in desirability trajectories, which was most pronounced when the faking impact was high and the model included a faking dimension.^6^

#### Recovery of latent correlations between substantive traits

In terms of the recovery of latent correlations between substantive traits, the above-described ANOVA framework (see Table S.I.1 for effect size estimates of all main effects and interactions) indicated that *Model* (bias: ${\eta}_{G}^{2}$ = .257; RMSE: ${\eta}_{G}^{2}$ = .452), *Item Desirability Characteristics* (bias: ${\eta}_{G}^{2}$ = .567; RMSE: ${\eta}_{G}^{2}$ = .272), and *Faking Impact* (bias: ${\eta}_{G}^{2}$ = .594; RMSE: ${\eta}_{G}^{2}$ = .733) had meaningful main effects on bias and RMSE. Again, there were two-way interactions (bias: .097 < ${\eta}_{G}^{2}$ s < .579; RMSE: .057 < ${\eta }_{G}^{2}$ s < .381) between these three factors that were qualified by a pronounced three-way interaction (bias: ${\eta}_{G}^{2}$ = .296; RMSE: ${\eta }_{G}^{2}$ = .165). All main effects and interactions associated with *Test Length*, *Sample Size*, and *Presence of Response Styles* were negligible (bias: all ${\eta}_{G}^{2}$ s < .025; RMSE: all ${\eta}_{G}^{2}$ s < .037).

**Fig. 3:**
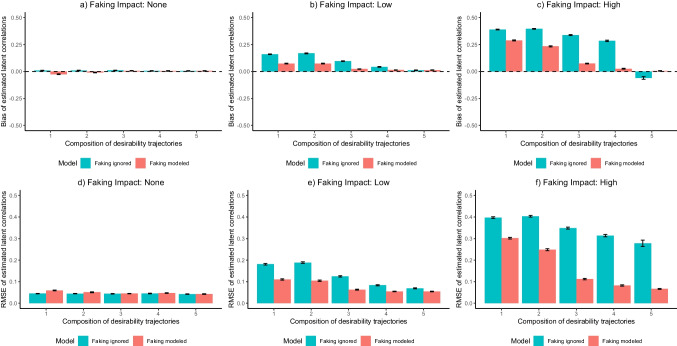
Simulation study: Recovery of latent correlations between substantive traits*. *The depicted recovery of latent correlations between substantive traits is for the representative case of six items per substantive trait scale, a sample size of 1000, and extreme response style (ERS) being present in the data. Models ignoring faking only included dimensions for substantive traits and ERS, whereas models accounting for faking also included a faking dimension. Values reflect the mean bias (*upper panel*) and root mean square error (RMSE; *lower panel*) across replications within a condition. Error bars represent the standard error of the mean

Hence, Fig. 3 shows the three-way interaction between *Model*, *Item Desirability Characteristics*, and *Faking Impact* for the representative case of 6 items per substantive trait scale, a sample size of 1000, and ERS being present in the data: When there was no faking in the data (see Fig. 3a, d), the estimation of latent correlations between substantive traits was unbiased in both the model ignoring faking and the model accounting for faking, irrespective of item desirability characteristics. RMSE also did not differ between the different models and item desirability characteristics. However, when there was a low faking impact (see Fig. 3b, e) and desirability trajectories of items were mainly increasing, estimated latent correlations were positively biased and had larger RMSE in the model ignoring faking, whereas the model accounting for faking attenuated the bias and increased precision. Along with the effect of the model, having more variety in desirability trajectories across items also led to a reduction of bias and RMSE. The same pattern occurred when the faking impact was high (see Fig. 3c, f), but with more pronounced effects. In this case, more variety in desirability trajectories across items only led to a complete elimination of bias and a considerable reduction of RMSE in models that accounted for faking. In models that ignored faking, latent correlations were biased and imprecisely recovered in all compositions of desirability trajectories.^7^

### Discussion of simulation results

The simulation results show that accounting for a faking dimension when modeling item responses that are potentially distorted by social desirability is worthwhile for estimating test-takers’ substantive trait levels as well as latent correlations between substantive traits. Results indicate that the extent to which modeling faking is superior to only modeling response styles such as ERS primarily depends on the impact of faking in the data. Effects were stronger when faking explained a large proportion of variance in item responses compared to when it only explained a small proportion or when it was absent. Importantly, even when faking was not part of the data-generating process, modeling faking was not associated with a worse estimation of substantive trait person parameters. That is, modeling faking with the MNRM does not erroneously attribute substantive trait variance to a faking dimension, which is a major limitation of SDR scales (e.g., de Vries et al., 2014; McCrae & Costa, 1983; Müller & Moshagen, 2019) and has been observed in the context of response styles (e.g., Merhof et al., 2023). Note that the findings were independent of the number of items per substantive trait scale, the number of test-takers, and the presence of ERS in the data. However, item desirability characteristics played an important role such that they moderated the effect of modeling faking and led themselves to different levels of recovery of substantive trait person parameters.

Regarding the recovery of latent correlations between substantive traits, the simulation yields a similar conclusion. Modeling faking led to less biased and more precise intercorrelations between substantive traits when faking was present in the data, particularly when the impact of faking was high. This indicates that modeling faking with the MNRM can indeed debias inflated correlations between substantive traits (e.g., Ellingson et al., 1999; Klehe et al., 2012; Schmit & Ryan, 1993) and thus facilitate nuanced test-taker profiles within a personality inventory. As for the recovery of substantive trait person parameters, the simulation findings were independent of test length, sample size, and presence of response styles, whereas having more variety in desirability trajectories across items interacted with the effect of modeling faking and could per se improve the recovery of latent correlations between substantive traits.

## Empirical demonstration

The simulation study had the purpose of investigating the potential of modeling faking with the MNRM when the data-generating process and true parameter values are known. To examine whether the faking modeling approach in combination with different item desirability characteristics also proves successful in empirical data, we collected questionnaire data using an experimental faking manipulation and a special set of items. To emulate that desirability depends on the social situation (e.g., Ellingson & McFarland, 2011; Kuncel & Tellegen, 2009) and that faking is inherent to the assessment context at hand, we used a specific social context for item responding, namely a hypothetical application for a leadership position in the industry.

### Development of items with different desirability characteristics

As noted by Peabody (1967) and other scholars (e.g., Bäckström et al., 2009; Wood et al., 2022), most personality items are constructed in a way that descriptive and evaluative aspects are confounded, that is, high rating scale categories are associated with both high substantive trait levels and high desirability levels. Hence, to examine the effects of different item desirability characteristics in empirical questionnaire data, we adapted items from a widely-used personality test, the German version of the *Big Five Inventory 2* (BFI-2; Danner et al., 2016, 2019), such that they should still measure the Big Five but deconfound substantive trait levels and desirability levels. That is, we modified the BFI-2 items to create more items with nonmonotonically increasing, inverted-U-shaped, and decreasing desirability trajectories. Note that this approach is different from the approach followed by Bäckström et al. (2009, 2023) and Wood et al. (2022, 2023), who merely aimed at reducing evaluative item content to counteract SDR instead of creating more variety in desirability trajectories. We then piloted the original and modified items to obtain empirical desirability ratings.

#### Steps of item modification

Before modifying items, we conducted a brief job demand analysis for a leadership position in the industry to get a better understanding of what is considered desirable and undesirable in this particular social context. We therefore looked for articles in the leadership literature portraying the role of different personality attributes (e.g., Ames & Flynn, 2007; Baron et al., 2000; De Hoogh et al., 2005; Hogan & Kaiser, 2005; Judge et al., 2002; Kaiser et al., 2015) and surveyed ten persons currently holding a leadership position. Based on the results of the job demand analysis, we then reworded items from the BFI-2 with the aim of creating modified items where higher rating scale categories are not monotonically related to higher desirability levels but where higher categories are still related to higher levels of the to-be-measured Big Five trait.^8^ Following certain strategies of item modification (see Supplement II for details), we created 104 modified items. In the next step, the modified items were reviewed regarding their fit to the construct definitions of the Big Five (McCrae & Costa, 1987) as well as regarding aspects like ambiguity and item length. This led to an exclusion of 39 modified items. The 60 original BFI-2 items as well as the 65 retained modified items can be found in Table A1 in the Appendix.

#### Pilot study

We then piloted the original and modified items to obtain empirical desirability ratings for the context of an application for a leadership position in the industry. Because we carried out the modification of items in two waves, we ran two piloting rounds to obtain desirability ratings for all 125 items. The study procedure and the population for participant sampling were, however, identical between the two piloting rounds. Forty-one modified items as well as the 60 BFI-2 items were piloted in the first round, the 24 remaining modified items in the second round.

##### Procedure

We ran the pilot study on the online data collection platform *SoSci Survey* (https://www.soscisurvey.de/). After giving informed consent and completing demographic measures, participants were asked to take the perspective of a person who is currently applying for a leadership position in the industry. We then familiarized participants with typical tasks of personnel in leadership positions before telling them that the application process would feature a questionnaire on personal attitudes and behaviors. Next, we informed them about their task: For every statement (i.e., item), they were instructed to judge which of seven graded agreement levels (i.e., response categories; 1 = *very low agreement* to 7 = *very high agreement*) is most desirable in the given context. Items were presented in a random order and on separate pages. After half of the items, participants could take a self-paced break. The exact instructions, data, as well as analysis code of the pilot study can be found at https://osf.io/ms57p/.

##### Sample

To obtain desirability ratings from people who could potentially apply for a leadership position, we allowed participation in the pilot study only if participants were at least 18 years old and already had work experience. Because the items were created in German, participants also needed to speak German fluently to be eligible for participation. We excluded participants from the analyses if they had failed at least one instructed-response item (e.g., “Please click here on scale point 3”), if they indicated that their data shall not be used, or if their median item response time was less than 50% of the median item response time across all participants (cf. Gummer et al., 2021; Leiner, 2019). The participant samples of the two piloting rounds had a similar distribution of age, gender, work experience, and leadership experience. The sample of the first round (*N* = 152) had a mean age of *M* = 28.43 years (*SD* = 13.06, range = [18–71]), with 71.1% being female (28.9% male). The mean work experience was *M* = 7.88 years (*SD* = 11.77, range = [1–41]), with the majority (85.5%) never having held a leadership position. The mean age in the sample of the second round (*N* = 196) was *M =* 26.01 years (*SD* = 11.20, range = [18–65]) and 73.0% were female (27.0% male). Participants in this sample had a mean work experience of *M* = 5.80 years (*SD* = 9.61, range = [1–49]) and 88.3% had never held a leadership position. The two samples did not differ significantly (*α* = .05) on any of the four demographic variables (|*t*s| < 1.87, *p*s > .062; *χ*^2^s < 0.36, *p*s > .552).

##### Results

After recoding desirability ratings for negatively-keyed items, we calculated Pearson’s *X*^2^ statistic for all desirability trajectory types *t* and each item *i* to classify the piloted items into the five desirability trajectory types that were also used in the simulation study:

4 $${X}_{ti}^{2}= {\sum\limits _{k=0}^{6}}\frac{{\left({O}_{ki}-{E}_{tk}\right)}^{2}}{{E}_{tk}}.$$

**Fig. 4:**
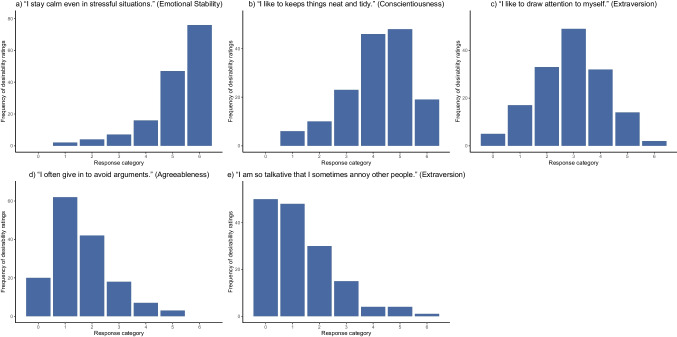
Empirical demonstration: Histograms of desirability ratings for five exemplary items*. N* = 152 for all of the five exemplary items. Classifications of desirability trajectories: **a**) monotonically increasing, **b**) nonmonotonically increasing, **c**) inverted-U-shaped, **d**) nonmonotonically decreasing, **e**) monotonically decreasing

*E*_*tk*_ denotes the expected frequency of desirability ratings of response category *k* under desirability trajectory type *t*. These values were derived from the prototypical desirability trajectories shown in Fig. 1 and the number of participants giving desirability ratings for the respective item. *O*_*ki*_ represents the observed frequency of desirability ratings of category *k* on item *i*. We then classified the items into the five desirability trajectory types based on the minimal *X*^2^ value that resulted for a given item. According to this classification, 38 (30.4%) of the 125 original and modified items had monotonically increasing desirability trajectories, 29 (23.2%) exhibited nonmonotonically increasing desirability trajectories, 32 (25.6%) inverted-U-shaped desirability trajectories, 19 (15.2%) nonmonotonically decreasing desirability trajectories, and 7 (5.6%) monotonically decreasing desirability trajectories. The classification of each item can be found in Table A1. Figure 4 shows histograms of desirability ratings for five exemplary items.

### Main study: Collecting item responses under low-stakes and high-stakes conditions

#### Design

After modifying the BFI-2 items and piloting them together with the original items concerning their desirability trajectories, we gave the whole item set to another sample of participants and instructed them to respond to the items under two conditions, namely an honest condition as well as a hypothetical application condition. The honest condition served as a low-stakes (LS) condition in which we asked participants to respond as honestly as possible. The hypothetical application condition served as an experimental high-stakes (HS) condition. In this condition, participants were instructed to respond as if they were applying for a leadership position in the industry. Also, they received a financial incentive to adapt their responses to meet the requirements of the advertised job. To control potential carry-over effects between the two conditions, we randomized the order of conditions between participants.

#### Procedure

*SoSci Survey* served as the online platform for data collection. At the beginning, participants were asked to give informed consent and complete demographic measures. Subsequently, they read the instructions of the first condition and responded to the original and modified items before reading the instructions of the second condition and responding again to all items. In the LS condition, we emphasized that there were no right or wrong answers and that the data were kept strictly confidential. In the HS condition, we asked participants to take the perspective of a person who is currently applying for a leadership position at a fictitious company in the industry. Therefore, participants saw a fictitious job advertisement for the vacant leadership position in which the company communicated tasks of their leadership personnel as well as their expectations about the personality of applicants. We then told participants that, in order to identify applicants who fit ideally to the vacant position, a questionnaire about personal attitudes and behaviors would be part of the application process. Subsequently, we instructed participants to respond to the items as if they were in the described application context. However, as is usually the case in high-stakes assessments, we asked them to respond based on their actual attitudes and behaviors, but at the same time try to get the vacant position. To create actual stakes for participants, we told them that the 10% of participants matching the personality profile from the job advertisement best would receive the double amount of the standard compensation for participation.^9^ The exact instructions from the two conditions can be found at https://osf.io/ms57p/. In both conditions, responses were given on a seven-point Likert scale (1 = *very low agreement* to 7 = *very high agreement*). In both conditions, items were presented in a randomized order and on separate pages. Participants had the opportunity to take a self-paced break after half of the items had been presented in each condition. However, before participants could respond to the items in a condition, they had to pass a diligence check item in which they were queried about how they were supposed to respond according to the instructions of the respective condition. After completing both conditions, participants were debriefed and thanked for participating. The completion of the entire study took approximately 30 minutes.

#### Sample

The sample of participants was collected via *Bilendi*, a European access panel service provider. Participants successfully completing the study received compensation worth 5 euros. As in the pilot study, we allowed participation only if participants were at least 18 years of age, already had work experience, and spoke German fluently. To ensure good data quality, we a priori implemented several quality checks in a way that participants failing at least one of these quality checks were immediately screened out and could not finish the data collection. In particular, we used the following quality checks (cf. Gummer et al., 2021; Leiner, 2019): two instructed-response items, the above-mentioned diligence check items querying participants about the preceding instructions, a self-report diligence check where participants could indicate that their data shall not be used, a longest-string analysis where participants were screened out if they provided at least ten identical responses to consecutive items in the LS condition, as well as a response time criterion where participants were screened out if their median item response time was less than 50% of the median item response time across the participants who had previously passed all quality checks. Because participants failing at least one of these quality checks were screened out during data collection, their data were not available for analysis and no post-hoc exclusion of participants needed to be made. The sample of participants passing all quality checks comprised *N* = 1070 subjects. Within this sample, the mean age was *M* = 36.78 years (*SD* = 13.06, range = [18–65]) and 54.0% were female (46.0% male). Regarding work and leadership experience, participants reported a mean work experience of *M* = 14.50 years (*SD* = 12.76, range = [1–53]) and 65.6% had never held a leadership position.

### Results of the empirical demonstration

Since the faking manipulation was operationalized within participants, it was possible to analyze item responses from one sample of test-takers in both an LS and HS condition. Considering the instructions as well as the financial incentive to distort responses in the HS condition, parameter estimates from models fitted to HS condition data could be expected to be systemically biased by faking. In contrast, given that participants were instructed to respond as honestly as possible in the LS condition and had no obvious motivation to present themselves in an overly favorable manner, parameter estimates from models fitted to LS condition data should not be systematically influenced by faking but represent approximations of true parameter values. This allowed us to compare the parameter estimates from the model ignoring faking and the model accounting for faking (both fitted to the HS condition data) regarding the question of which model represents the approximated true parameter values (i.e., estimates from the LS condition data) better.

#### Composition of item sets with different desirability trajectories

To be able to examine the effects of different item desirability characteristics, we composed five item sets mirroring the five compositions of desirability trajectories from the simulation study (see Fig. 1). Each item set consisted of 30 items, with 6 items per Big Five trait. Descriptively, the desirability trajectory type of an item was strongly related to the mean shift of item responses between the LS and HS condition (polyserial correlation of .84, *p* < .001): Items with monotonically increasing desirability trajectories had an item mean that was on average 0.74 scale points higher in the HS than in the LS condition, whereas items with nonmonotonically increasing desirability trajectories yielded a mean shift of 0.56, items with inverted-U-shaped desirability trajectories a mean shift of 0.31, items with nonmonotonically decreasing desirability trajectories a mean shift of –0.27, and items with monotonically decreasing desirability trajectories a mean shift of –0.58. To compose the five item sets in the proportions of desirability trajectories as indicated in Fig. 1, we selected the items that measured the underlying Big Five trait best. Therefore, we fitted a model with all 125 items to the LS condition data, in which only substantive traits and ERS were accounted for (Bolt & Newton, 2011; Wetzel & Carstensen, 2017). In this model, we considered the estimated item slopes to select the items with highest discrimination concerning the underlying Big Five trait in a dataset where item responses should not be systematically influenced by faking. To ensure that the meaning of the substantive traits as measured in the BFI-2 did not fundamentally change when adding the modified items, we fixed the slopes of the BFI-2 items to the estimated values from a corresponding model in which only the 60 BFI-2 items were modeled in the LS condition data. The five item sets that were composed based on this procedure as well as reliability estimates and convergent validities with the original BFI-2 can be found in Table S.II.1 in Supplement II.

#### Fitted models

Within each item composition, we fitted different models to the HS condition data. We used the MH-RM algorithm implemented in the R package *mirt*, imposed model identification constraints as described above, and estimated person parameters via MAP scores. In particular, we fitted a model only accounting for substantive traits, a model accounting for substantive traits and ERS, as well as a model additionally accounting for faking. As in the simulation, we linearly transformed the scoring weights of the ERS and faking dimensions to a range from 0 to 6 for a common metric of scoring weights across all dimensions (cf. Falk & Ju, 2020). Such linear transformations of scoring weights do not affect the estimation of person parameters, latent correlations, and model fit. Scoring weights of the faking dimension were based on the relative frequencies of desirability ratings from the pilot study, which can be found in Table S.II.1.

All models converged within 718 MH-RM iterations. Table 2 contains absolute (Cai & Monroe, 2013, 2014; Maydeu-Olivares & Joe, 2014) and relative model fit measures for the fitted models. In all compositions of desirability trajectories, fit measures consistently indicated that modeling ERS improved model fit compared to only modeling substantive traits. Crucially, adding a faking dimension improved model fit further in all item compositions, showing that the faking modeling approach could explain incremental variance in item responses over and above response styles.

**Table 2:** Empirical demonstration: Model fit measures of fitted models

	Absolute fit measures				Relative fit measures			
Dimensions modeled	*C*_2_ (*df*), *p*-value	RMSEA	CFI	TLI	Log-likelihood	AIC	BIC	LR test
*Composition of desirability trajectories 1:*								
θs	3059.9 (395), *p* < .001	.079	.947	.952	–40815.2	82070.5	83165.1	
θs/ERS	2687.2 (360), *p* < .001	.078	.949	.958	–38850.4	78210.9	79479.6	*χ*^2^(35) = 3929.6, *p* < .001
**θs/ERS/Faking**	**1537.3 (324), *p* < .001**	**.059**	**.971**	**.978**	**–38459.1**	**77500.2**	**78948.0**	***χ***^**2**^**(65) = 782.7, *p* < .001**
*Composition of desirability trajectories 2:*								
θs	2444.2 (395), *p* < .001	.070	.942	.947	–44691.3	89822.5	90917.1	
θs/ERS	2166.1 (360), *p* < .001	.069	.944	.954	–42857.0	86224.0	87492.7	*χ*^2^(35) = 3668.5, *p* < .001
**θs/ERS/Faking**	**1684.1 (324), *p* < .001**	**.063**	**.953**	**.965**	**–42688.9**	**85959.8**	**87407.7**	***χ***^**2**^**(36) = 336.1, *p* < .001**
*Composition of desirability trajectories 3:*								
θs	3186.7 (395), *p* < .001	.081	.896	.906	–47609.5	95659.1	96753.7	
θs/ERS	2882.4 (360), *p* < .001	.081	.897	.915	–45723.5	91956.9	93225.7	*χ*^2^(35) = 3772.2, *p* < .001
**θs/ERS/Faking**	**2100.9 (324), *p* < .001**	**.072**	**.919**	**.940**	**–45493.3**	**91568.5**	**93016.4**	***χ***^**2**^**(36) = 460.4, *p* < .001**
*Composition of desirability trajectories 4:*								
θs	3900.6 (395), *p* < .001	.091	.787	.807	–51303.0	103046.0	104140.6	
θs/ERS	2575.2 (360), *p* < .001	.076	.853	.878	–49235.7	98981.4	100250.1	*χ*^2^(35) = 4134.6, *p* < .001
**θs/ERS/Faking**	**1614.8 (324), *p* < .001**	**.061**	**.905**	**.929**	**–48897.5**	**98377.0**	**99824.8**	***χ***^**2**^**(36) = 676.4, *p* < .001**
*Composition of desirability trajectories 5:*								
θs	3716.8 (395), *p* < .001	.089	.797	.816	–52092.2	104624.5	105719.1	
θs/ERS	2275.6 (360), *p* < .001	.071	.872	.894	–49828.4	100166.7	101435.5	*χ*^2^(35) = 4527.7, *p* < .001
**θs/ERS/Faking**	**1486.3 (324), *p* < .001**	**.058**	**.914**	**.936**	**–49496.2**	**99574.5**	**101022.3**	***χ***^**2**^**(36) = 664.3, *p* < .001**

*Note. N* = 1070. The five compositions of desirability trajectories correspond to those displayed in Fig. 1. Models were fitted to data from the high-stakes (HS) condition. *C*_2_ = limited information fit statistic *C*_2_; RMSEA = root mean square error of approximation; CFI = comparative fit index; TLI = Tucker-Lewis index; AIC = Akaike information criterion; BIC = Bayesian information criterion; LR test = likelihood-ratio test (here: hierarchical comparison of nested models); ERS = extreme response style. The best-fitting model within each item composition is printed in bold

#### Correlations between low-stakes and high-stakes substantive trait person parameters

Assuming that person parameters of substantive traits in the LS condition were not systematically biased by faking, we looked at correlations of substantive trait person parameters between the LS and HS condition to examine if the model including a faking dimension could recover the substantive trait person parameters from the LS condition better than a model not accounting for faking. Modeling ERS yielded a significantly better model fit than not accounting for response styles in all item compositions also in the LS condition data (*χ*^2^s(35) > 3390.4, *p*s < .001). Hence, we computed correlations of substantive trait person parameters from the model accounting for substantive traits and ERS in the LS condition a) with person parameters from the corresponding model in the HS condition and b) with person parameters from the model additionally accounting for faking in the HS condition. We compared these two correlations for all Big Five traits in the five item compositions.

**Fig. 5:**
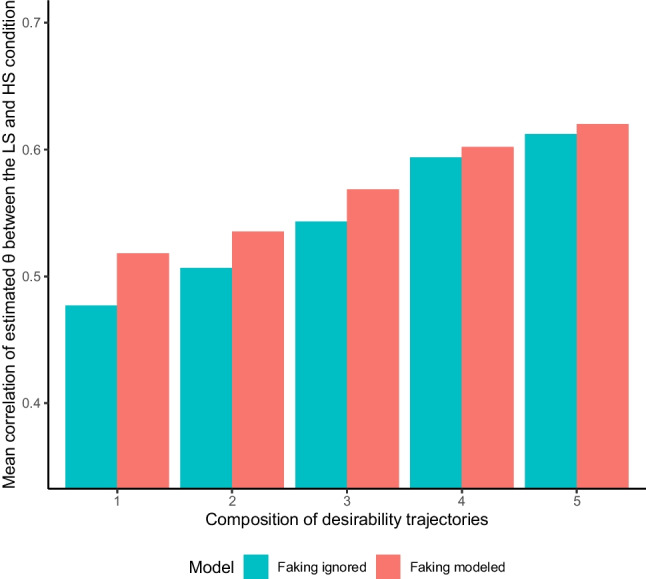
Empirical demonstration: Mean correlations of substantive trait person parameters between the low-stakes and high-stakes condition*. *The depicted mean correlations are aggregated across the Big Five and reflect the back-transformed means of the Fisher-*z*-transformed correlations of substantive trait person parameters between the low-stakes (LS) and high-stakes (HS) condition. To the LS condition data, a model ignoring faking was fitted, whereas a model ignoring faking and a model accounting for faking were fitted to the HS condition data. The five compositions of desirability trajectories correspond to those displayed in Fig. 1. Models ignoring faking only included dimensions for substantive traits and ERS, whereas models accounting for faking also included a faking dimension

For 22 of the 25 comparisons, person parameters from the LS condition were descriptively more strongly associated with person parameters from the HS condition when faking was modeled in the HS condition data than when faking was not modeled. The difference in correlations reached significance (*α* = .05, one-tailed tests) for 19 of the 25 comparisons. All correlations and significance tests can be found in Table S.II.2 in Supplement II. Figure 5 shows the mean correlations between the LS and HS condition across the Big Five traits for the model ignoring faking and the model accounting for faking in the five item compositions. Along with the overall higher correlations for the model accounting for faking, it is noticeable that differences in correlations were more pronounced in item compositions that predominantly consisted of items with increasing desirability trajectories as compared to item compositions that also contained items with decreasing desirability trajectories. Moreover, the latter item compositions yielded generally higher correlations of person parameters than the former item compositions. This pattern of results mirrors the result pattern from the simulation study for the case of a low faking impact (see Fig. 2).

#### Latent correlations between substantive traits

**Fig. 6:**
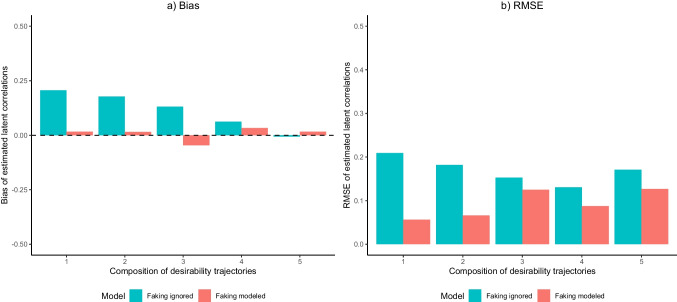
Empirical demonstration: Bias and root mean square error (RMSE) of estimated latent correlations between substantive traits*. *The depicted values refer to the mean bias and root mean square error (RMSE) of estimated latent correlations between substantive traits in the high-stakes (HS) condition with respect to the latent correlations from a model ignoring faking in the low-stakes (LS) condition. To the LS condition data, a model ignoring faking was fitted, whereas a model ignoring faking and a model accounting for faking were fitted to the HS condition data. The five compositions of desirability trajectories correspond to those displayed in Fig. 1. Models ignoring faking only included dimensions for substantive traits and ERS, whereas models accounting for faking also included a faking dimension

Under the assumption that item responses in the LS condition were not systematically affected by faking, the estimated latent correlations between substantive traits in the LS condition should serve as unbiased approximations of the intercorrelations between the substantive traits as measured in the present items. To examine the effects of modeling faking as well as the different item desirability characteristics on the estimation of latent correlations between substantive traits in the HS condition, we calculated the mean bias and RMSE of the estimated latent correlations between the Big Five with respect to the corresponding latent correlations from the model accounting for substantive traits and ERS in the LS condition. Results are displayed in Fig. 6.

Regarding bias, the model accounting for faking reduced the bias of latent correlations compared to the model ignoring faking in item compositions that contained items with increasing desirability trajectories. When there were also items with inverted-U-shaped desirability trajectories, the model accounting for faking induced a slight negative bias, whereas latent correlations in the model ignoring faking were still considerably positively biased. When decreasing desirability trajectories were present, latent correlations in both models were almost unbiased. This pattern generally mirrors the findings from the simulation (see Fig. 3). Concerning RMSE, latent correlations in the model accounting for faking had considerably smaller RMSE than latent correlations in the model ignoring faking when items only had increasing desirability trajectories, which is in line with the simulation findings. However, unlike in the simulation, more variety in desirability trajectories across items was not associated with smaller RMSE in the model accounting for faking. However, when item compositions also contained inverted-U-shaped and/or decreasing desirability trajectories, RMSE in the model accounting for faking was still smaller than in the model ignoring faking.

### Discussion of results of the empirical demonstration

The purpose of the empirical demonstration was to investigate if the MNRM approach to modeling faking in combination with different item desirability characteristics also proves successful in empirical questionnaire data. Replicating the findings from Seitz et al. (2023), the results show that modeling faking can improve model fit over and above modeling response styles in experimental high-stakes assessment data.

Importantly, the results also demonstrate that modeling faking can overall increase the extent to which low-stakes substantive trait person parameters, which serve as benchmarks of trait assessment not influenced by deliberate faking, are recovered in high-stakes data. Note that this was found although the model in the LS condition and the model ignoring faking in the HS condition had the same dimensional structure (namely, five substantive traits and ERS), whereas the model accounting for faking additionally included a faking dimension. Moreover, effects were moderated by item desirability characteristics such that the effect of modeling faking was most pronounced in item compositions with predominantly increasing desirability trajectories. In item compositions also containing decreasing desirability trajectories, the effect of the modeling was practically negligible. Additionally, item compositions with more variety in desirability trajectories were associated with generally better recovery of low-stakes person parameters in high-stakes data, irrespective of whether faking was modeled.

Regarding latent correlations between substantive traits, the results also replicate Seitz et al.’s (2023) findings to the effect that adding a faking dimension to the model can debias inflated latent correlations between substantive traits and increase the precision of estimation. Like for substantive trait person parameters, the results indicate that more variety in desirability trajectories across items can have a debiasing effect regardless of whether or not the model contains a faking dimension. Estimation precision, however, did not consistently improve with more variety in desirability trajectories across items.

Overall, the findings of the empirical demonstration are aligned with the simulation results. Concerning both the pattern of results and the relatively small effect sizes concerning the estimation of substantive trait person parameters, the findings are especially in line with the case of a low faking impact in the data (see Figs. 2 and 3). This constitutes a plausible finding considering the experimental nature of the present HS condition, in which all participants received the same explicit instruction to respond based on their actual attitudes and behaviors but try to get the vacant position at the same time. It is possible that the strong situational cues in the experimental setting, in which no strong differences in the motivation to adhere to the instructions can be expected, led to restricted variation in the degree of faking between participants (cf. Birkeland et al., 2006; McFarland & Ryan, 2000), implying a relatively low impact of a latent faking dimension. Psychometrically, this is reflected in the estimated slopes of the faking dimension, which were on average notably smaller ($\overline{\alpha}$_⋅Faking_ = 0.12) than the estimated slopes of the substantive trait dimensions ($\overline{\alpha}$_⋅θs_ = 0.80).

## General discussion

In the present research, we applied IRT modeling to account for the response bias of faking. Using a simulation and an empirical demonstration, we investigated under which circumstances the MNRM approach to modeling faking can adequately adjust substantive trait scores and latent correlations between substantive traits for the influence of faking. In particular, we were interested in how different item desirability characteristics can facilitate the modeling of faking and counteract its detrimental effects.

### Utility of modeling faking

As outlined in the introduction, the faking modeling approach of this article entails an item-dependent specification of how response categories are related to social desirability and thus allows for a confirmatory modeling of faking. In contrast, approaches that aim to account for faking in a data-driven manner (e.g., exploratory mixture models with latent faking classes; Zickar et al., 2004) do not directly justify the assertion that it is faking and not an unknown combination of other response biases that has been accounted for. Furthermore, the model allows for curvilinear relationships between response categories and social desirability (cf. Kuncel & Tellegen, 2009) because scoring weights of faking are specified in an item- and category-specific manner. This constitutes an important advantage over other recent latent variable models of faking (e.g., Böckenholt, 2014; Brown & Böckenholt, 2022; Hendy et al., 2021; Leng et al., 2020; Ziegler & Bühner, 2009), which do not explicitly account for item-specific trajectories of social desirability over response categories and hence neglect this relevant information in item responses.

Overall, we found that the adjustments of substantive trait person parameters afforded by the MNRM including a faking dimension are indeed associated with a more accurate representation of test-takers’ substantive trait levels compared to only accounting for substantive traits and ERS in high-stakes assessment data (see LaHuis et al., 2019; Sun et al., 2022). Thus, modeling faking contributes to a purer assessment of interindividual differences. From an applied perspective, this can enhance test fairness and can help that decisions in high-stakes contexts, such as hiring decisions in personnel selection, are based on substantive trait score estimates that more closely reflect the traits of interest. Also, we found that latent correlations between substantive traits are debiased and more precisely estimated when faking is accounted for in the model. In applied settings, this implies that more valid conclusions on relationships between the assessed traits can be drawn and that, given that correlations between generally desirable traits are usually inflated in high-stakes assessments, more nuanced test-taker profiles within a personality inventory are possible.

Furthermore, the simulation showed that the extent to which a model including a faking dimension is superior to a model not including a faking dimension strongly depends on the impact of faking in the data. Unsurprisingly, a model with faking dimension outperformed a model without faking dimension more strongly when the faking impact in the data-generating process was high than when it was low. Crucially, however, a model with faking dimension was never inferior to a model without faking dimension and did not erroneously attribute substantive trait variance to a faking dimension, even when faking was completely absent in the data. Thus, using the model with faking dimension can be recommended in applied contexts in which faking might occur.

### Importance of item desirability characteristics

Along with studying the mere psychometric benefits of the faking modeling approach, we examined the effects of different desirability characteristics of items. That is, we investigated how variation in the way item content is related to social desirability can facilitate the modeling of faking and counteract its detrimental effects. As Peabody (1967) noted, most personality items confound descriptive aspects with evaluative aspects, which implies that high scores can be due to a high substantive trait level, a high faking tendency, or both, unless faking is statistically accounted for. Concerning the MNRM, however, the confound between descriptive and evaluative aspects causes high collinearity between the scoring weight vectors of the substantive trait and faking dimensions, which arguably makes it difficult to properly disentangle substantive traits and faking.

In the simulation, it turned out that item desirability characteristics, on the one hand, moderate the effect of modeling faking and, on the other hand, have a main effect regarding the recovery of substantive trait person parameters and latent correlations between substantive traits. In particular, a model accounting for faking was differentially superior to a model ignoring faking depending on the composition of desirability trajectories. Additionally, more variety in desirability trajectories across items was associated with a generally better parameter recovery.

When the faking impact was low, the difference in parameter recovery between a model ignoring faking and a model accounting for faking was most pronounced in item compositions that only contained increasing desirability trajectories. Hence, despite collinearity between scoring weight vectors of substantive traits and faking in these item compositions, modeling faking can be particularly worthwhile compared to ignoring faking. Nevertheless, parameter recovery in item compositions with mainly increasing desirability trajectories was generally worse than in item compositions that also contained decreasing desirability trajectories. Here, a model ignoring faking could also recover parameters well. For the case of a low faking impact, the effects of faking on item responses hence seem to cancel each other out across the items of a test with balanced desirability trajectories, such that even ignoring faking can yield satisfactory results.

However, when the faking impact was high, modeling faking was notably superior to not modeling faking in all item compositions. Again, more variety in desirability trajectories was associated with a generally better parameter recovery, but a model without faking dimension only profited considerably from this in item compositions that contained all desirability trajectory types. A model with faking dimension, in contrast, entailed good parameter recovery even if increasing desirability trajectories were complemented only with inverted-U-shaped and/or nonmonotonically decreasing desirability trajectories. Hence, for the case of a high faking impact, modeling faking is necessary to achieve good parameter recovery irrespective of item desirability characteristics. Conceptually, effects of faking on item responses can also be expected to cancel each other out across items with balanced desirability trajectories when the faking impact is high. Because the effects of faking on item responses do not need to be constant across items, however, it is unlikely that the effects average out entirely within a given item set. Considering that this imperfect averaging-out should be more pronounced in case of a high faking impact, a model that accounts for item-specific effects of faking is required irrespective of item desirability characteristics to recover parameters well in this case.

Having modified items from the widely-used personality test BFI-2, we also demonstrated that it is possible to create more variety in empirical desirability trajectories through item refinement and that this is associated with the same result patterns as in the simulation. Hence, deconfounding descriptive and evaluative aspects in items of a personality test is not only appealing from a theoretical and conceptual point of view but is also feasible empirically and has utility for applied assessments. Resembling the findings from the simulation study, item desirability characteristics in the empirical demonstration interacted with the effect of modeling faking and had a main effect regarding the extent to which the estimates from the HS condition recovered the LS condition estimates. Despite collinearity between scoring weight vectors of substantive traits and faking, the effect of modeling faking was most pronounced in item compositions that only consisted of items with increasing desirability trajectories. More variety in desirability trajectories, in turn, reduced differences between modeling faking and ignoring faking, but led to an estimation of parameters that generally better recovered the LS condition estimates, mirroring the simulation findings from conditions with a low faking impact. As discussed above, this stands to reason considering the experimental nature of the present faking manipulation.

Note, however, the differences between the item modification approach of this article and other approaches of item redesign.^10^ Bäckström et al. (2009, 2023) and Wood et al. (2022, 2023), for example, followed an approach of neutralizing item evaluativeness. Like our approach, this approach aims to reword items regarding desirability but preserve the substantive item content. However, whereas our approach seeks to create variation in desirability trajectories across items, the approach of item evaluativeness neutralization implies that either all response categories have the same (intermediate) level of desirability or that the midpoint of the rating scale has the highest desirability level. Items modified according to this approach are, however, not suited for modeling faking by means of the MNRM because they either have constant faking scoring weights for all categories or have scoring weight vectors of faking that are redundant to scoring weight vectors of other response biases. The former would make the faking dimension irrelevant, the latter would make it impossible to separate faking from response styles like midscale response style (MRS), which is the tendency to prefer the midpoint category of a rating scale irrespective of item content.

One might argue that neutralizing item evaluativeness can eliminate SDR and faking in the first place. However, it is questionable whether test-takers would indeed only respond according to their substantive traits once they are confronted with an evaluatively neutral item. Apart from the fact that items with reduced evaluativeness can still give test-takers information regarding desirability (Wood et al., 2023), it is likely that, even for perfectly neutral items, test-takers would still try to figure out what is desirable in the given assessment context and then edit responses according to their idiosyncratic conclusions. In turn, by modeling items with pretested desirability trajectories, the MNRM explicitly takes item-specific response editing into account and thus affords a model-based separation of substantive traits and faking. Additionally, it yields estimates of each test-taker’s degree of faking in a given assessment context. Having such an estimate can be a helpful piece of information to evaluate the trustworthiness of responses from a test-taker in an applied assessment and can be used to study the substantive nature of the faking construct (Seitz et al., 2023).

### Limitations

Along with the above-described advantages of modeling faking by means of the MNRM in combination with different item desirability characteristics, some limitations should be mentioned. First, the fact that faking scoring weights for a specific item set and assessment situation are not readily transferable to other items or another assessment context can be considered a pragmatic limitation because additional resources will be required to adequately specify faking scoring weights if one wants to model different items or responses from different assessment settings. At the same time, the specificity of faking scoring weights can also be regarded as an asset of the present modeling approach because the modeling of faking is thus tailored to the specific assessment situation at hand.

Second, because scoring weights of faking are person-invariant model parameters, it is implicitly assumed that one desirability trajectory per item is appropriate to capture faking for all test-takers. However, as can be seen in the variability of desirability ratings in the pilot study, people do not perfectly agree about the most desirable category of an item. The more strongly test-takers differ in how they perceive desirability and respond according to it, the less appropriate it will be to use scoring weights of faking that are fixed across persons. To incorporate individual desirability perceptions of test-takers, however, one would have to collect additional data from the same test-takers whose actual item responses are to be modeled, which has multiple methodological shortcomings and will often not be possible in practice. Instead, the model makes the assumption that individual deviations in desirability perceptions from the specified item desirability characteristics are unsystematic fluctuations around a desirability trajectory that is on average representative for all test-takers. Thus, the model uses average desirability perceptions concerning each item to account for faking along with substantive traits and other response biases. This extends previous faking modeling approaches that assume effect patterns of faking to be constant across both persons and items (e.g., Böckenholt, 2014; Brown & Böckenholt, 2022; Hendy et al., 2021; Leng et al., 2020; Ziegler & Bühner, 2009). Nevertheless, to keep systematic deviations between the specified desirability characteristics and test-takers’ real desirability perceptions minimal, we advise researchers and practitioners to collect desirability ratings from a pilot study sample that is maximally similar to the sample of actual test-takers in terms of demographic features and contextual factors. Also, future research should investigate how much disparity in test-takers’ desirability perceptions is acceptable for the presented faking modeling approach to produce satisfactory results. For such robustness checks of the model, further simulation studies would be appropriate to determine a criterion for the necessary level of agreement in individual desirability perceptions. At the same time, if one knows about systematic deviations of desirability perceptions between groups of test-takers (e.g., young professionals vs. experienced hires), future studies could also specify scoring weights of faking group-specifically.

Third, modifying items to create more variety in desirability trajectories can be a challenging endeavor for items of substantive traits that are inherently desirable or undesirable in a given context. As Wood et al. (2022) noted, social desirability of personality items can be “partially intrinsic and partially the result of item writing practices” (p. 818). That is, for personality traits that are intrinsically desirable (undesirable), it can be hard to generate items with inverted-U-shaped and/or decreasing (increasing) desirability trajectories and, at the same time, not change the meaning of the assessed constructs. The risk of subtly changing the meaning of the construct applies to all kinds of item rewording, but especially to the attempt of creating items with decreasing (increasing) desirability trajectories for personality traits that intrinsically intertwine substantive and desirable (undesirable) attributes. To meet this problem, it is vital to review modified items regarding their fit to the construct definitions and to only include items in the final test form that still discriminate well concerning the traits of interest. In the empirical demonstration of this article, we did so by selecting the items with highest discrimination concerning the underlying Big Five trait in the condition in which participants were instructed to respond honestly. Nevertheless, as can be seen in Table S.II.1, convergent validities with the BFI-2 dropped to some extent in item compositions that also contained decreasing desirability trajectories compared to item compositions that only comprised increasing desirability trajectories. Specifically, the correlations between the sum score from the original BFI-2 items and the sum scores from the five item compositions ranged from .94 to .75 for Extraversion, from .92 to .65 for Agreeableness, from .97 to .63 for Conscientiousness, from .95 to .72 for Emotional Stability, and from .93 to .84 for Openness. However, these values are still in the upper range of typical convergent validities between different Big Five tests that feature distinct facets and emphases. Danner et al. (2019), for instance, reported correlations between the BFI-2 and other popular Big Five tests ranging from .88 to .64. Soto and John (2017) similarly found convergent validities ranging from .94 to .68. Thus, in the empirical demonstration of this article, changes in convergent validities associated with the modification of item desirability characteristics were empirically no larger than must be expected when switching from one Big Five test to another. Concerning applied settings, we argue that it mainly depends on the researcher’s or practitioner’s goals how much change in the meaning of the construct can be accepted. In personality research contexts, where the primary goal is to measure personality traits that are narrowly defined by particular facets, modifying item desirability characteristics might be less appropriate than in applied measurement contexts, such as high-stakes assessments in personnel selection, where the primary goal is to have a fair assessment that is not contaminated by faking. The more important this latter goal is, the more will subtle changes in the construct meaning be offset by having a measure that is not easily fakable, especially if the scoring of the test is based on the presented IRT model where different item desirability characteristics are explicitly modeled by the faking dimension.

### Future research directions

In the faking modeling approach presented in this article, faking is conceptualized as a continuous individual difference variable. Even though treating faking as such an individual difference variable is consistent with Ziegler et al.’s (2015) finding that faking mainly represents a continuous variable as opposed to a manifestation of distinct response processes, there might be heterogeneity in response strategies over and above quantitative variation in the degree of faking. To further examine the nature of heterogeneity in faking, future research could extend the model of this article in a mixture modeling framework by allowing for latent classes characterized by qualitatively different response processes. Also, faking might be better described by distributions other than a normal distribution. Future studies could, for instance, model faking using a truncated normal or log-normal distribution. This would correspond to a conceptualization of faking as a unipolar construct. Recent IRT approaches for unipolar modeling of performance data or psychopathological constructs (e.g., Huang & Bolt, 2023; Lucke, 2015) could be used as a starting point for future model extensions in this regard, though such models are currently limited to the case of modeling a single latent dimension.

Follow-up studies could also examine how the approach of changing item desirability characteristics affects the prediction of outcomes that are of interest in high-stakes assessments, such as job performance. Different effects are conceivable: First, considering that studies have often found a limited influence of SDR and faking on predictive validity (e.g., Ones et al., 2007; Paunonen & LeBel, 2012), it could be that correlations with outcomes are not affected. Second, given that part of the desirable item content can be beneficial for predicting performance outcomes (e.g., Li & Bagger, 2006; Wood et al., 2023), it could be that the prediction of these outcomes deteriorates because the modified item sets also capture less desirable aspects of the traits of interest. Third, it could be that the prediction improves, assuming that faking acts as a suppressor in predicting outcomes (e.g., Bing et al., 2011; Hakstian & Ng, 2005) and that substantive trait scores are less distorted by faking once there is more variety in desirability trajectories.

Moreover, since the current study featured an experimental faking manipulation, future studies should replicate the results in high-stakes assessment data from the field, for instance, in a dataset that contains responses from the same test-takers as job applicants and as job incumbents. However, compared to other studies in which faking was induced experimentally, the present study aimed at approximating the circumstances of an actual application context in two ways: First, the faking manipulation in this study instructed participants to base responses on their actual attitudes and behaviors (which is a typical response instruction in a personnel selection context) but at the same time try to get the vacant position (which is the goal of people applying for a particular job). In the faking literature, however, it is not uncommon to find blatant faking instructions in which participants are simply told to respond in a socially desirable manner. Second, there was a financial incentive for response distortion that created actual stakes for participants. Assessment results thus carried real consequences, emulating the incentive structure of a high-stakes testing like in personnel selection (i.e., a dilemma between sticking to the instruction of responding honestly and giving distorted responses to receive the reward). Nonetheless, follow-up studies with data from actual personnel selection contexts would be welcome. Thereby, it would also be appealing to further validate the model’s adjustments of substantive trait scores with personality measures that are less susceptible to faking, such as multidimensional forced-choice (MFC) measures (e.g., Cao & Drasgow, 2019) or observer ratings of personality (e.g., König et al., 2017).

### Conclusion

To conclude, the present research demonstrates two interacting approaches to address the response bias of faking: First, the MNRM provides an appealing framework for statistically modeling the influence of faking on item responses, which is particularly effective when the faking impact in the data is high. Second, modifying desirability characteristics of items can be a means to facilitate the modeling of faking and to counteract its adverse effects in the first place. Furthermore, this article highlights circumstances under which a statistical modeling of faking is particularly important and useful to improve the assessment of psychological constructs, and it reveals the beneficial effects of considering item desirability characteristics already at the stage of item construction to remedy the negative psychometric effects of faking. Our findings provide guidelines for applied researchers and practitioners to decide when using the MNRM to model faking is worthwhile and how to address faking by refining self-report personality questionnaires.

## Electronic supplementary material

Below is the link to the electronic supplementary material.

Supplementary file1 (DOCX 1.07 MB)

Supplementary file2 (DOCX 106 KB)

## Data Availability

All data and materials are available at https://osf.io/ms57p/.

## Appendix: List of items used in the empirical demonstration

**Table A1:** BFI-2 and modified items with their respective desirability trajectory classification

Item code	German version	English translation	Desirability trajectory classification
*BFI-2 items, Extraversion:*			
BFI_E01	Ich gehe aus mir heraus, bin gesellig.	I am outgoing, sociable.	nonmonotonically increasing
BFI_E02	Ich bin eher schüchtern. (R)	I am rather shy. (R)	monotonically increasing
BFI_E03	Ich bin eher ruhig. (R)	I am rather quiet. (R)	inverted-U-shaped
BFI_E04	Ich bin gesprächig.	I am talkative.	nonmonotonically increasing
BFI_E05	Ich bin durchsetzungsfähig, energisch.	I am assertive, energetic.	monotonically increasing
BFI_E06	Ich neige dazu, die Führung zu übernehmen.	I tend to act as a leader.	monotonically increasing
BFI_E07	Mir fällt es schwer, andere zu beeinflussen. (R)	I find it hard to influence people. (R)	nonmonotonically increasing
BFI_E08	In einer Gruppe überlasse ich lieber anderen die Entscheidung. (R)	In a group, I prefer to have others take charge. (R)	monotonically increasing
BFI_E09	Ich schäume selten vor Begeisterung über (R).	I rarely feel excited or eager. (R)	nonmonotonically increasing
BFI_E10	Ich bin weniger aktiv und unternehmungslustig als andere. (R)	I am less active and adventurous than other people. (R)	nonmonotonically increasing
BFI_E11	Ich bin voller Energie und Tatendrang.	I am full of energy and drive.	monotonically increasing
BFI_E12	Ich bin begeisterungsfähig und kann andere leicht mitreißen.	I am enthusiastic and can easily carry others along.	monotonically increasing
*Modified items, Extraversion:*			
mod_E01	Ich brauche ständigen Kontakt zu anderen Menschen.	I need constant contact with other people.	inverted-U-shaped
mod_E02	Mir fällt es leicht, auch einmal zu schweigen. (R)	It is easy for me to remain silent once in a while. (R)	nonmonotonically decreasing
mod_E03	Ich verwickle andere gerne in sehr lange Gespräche.	I like to engage others in very long conversations.	nonmonotonically decreasing
mod_E04	Ich bin geschwätzig.	I am chatty.	nonmonotonically decreasing
mod_E05	Ich bin so redselig, dass ich anderen damit manchmal auf die Nerven gehe.	I am so talkative that sometimes I annoy other people.	monotonically decreasing
mod_E06	Ich stehe ungern im Mittelpunkt des Interesses. (R)	I don’t like to be the center of interest. (R)	nonmonotonically increasing
mod_E07	Ich ziehe gerne die Aufmerksamkeit auf mich.	I like to draw attention to myself.	inverted-U-shaped
mod_E08	Bei Gruppenprojekten stehe ich meistens nicht im Mittelpunkt. (R)	I am usually not the center of attention in group projects. (R)	nonmonotonically increasing
mod_E09	Für gewöhnlich dominiere ich Gespräche.	I usually dominate conversations.	inverted-U-shaped
mod_E10	Ich kann Freude daran haben, nicht aktiv zu sein. (R)	I can find joy in not being active. (R)	nonmonotonically increasing
mod_E11	In einer Gruppe bin ich bei jeder Aktivität dabei.	In a group, I participate in every activity.	inverted-U-shaped
mod_E12	Mein Tatendrang überfordert andere manchmal.	My drive for action sometimes overwhelms others.	inverted-U-shaped
mod_E13	Mit meiner Begeisterung schieße ich gelegentlich über das Ziel hinaus.	I occasionally overshoot the mark with my enthusiasm.	inverted-U-shaped
*BFI-2 items, Agreeableness:*			
BFI_A01	Ich bin einfühlsam, warmherzig.	I am compassionate, warm-hearted.	nonmonotonically increasing
BFI_A02	Ich habe mit anderen wenig Mitgefühl. (R)	I have little sympathy for others. (R)	monotonically increasing
BFI_A03	Ich bin hilfsbereit und selbstlos.	I am helpful and unselfish with others.	nonmonotonically increasing
BFI_A04	Andere sind mir eher gleichgültig, egal. (R)	Others are of no concern and inconsequential to me. (R)	monotonically increasing
BFI_A05	Ich begegne anderen mit Respekt.	I treat others with respect.	monotonically increasing
BFI_A06	Ich habe oft Streit mit anderen. (R)	I often have arguments with others. (R)	monotonically increasing
BFI_A07	Ich bin manchmal unhöflich und schroff. (R)	I am sometimes rude and harsh. (R)	monotonically increasing
BFI_A08	Ich bin höflich und zuvorkommend.	I am polite and courteous.	monotonically increasing
BFI_A09	Ich neige dazu, andere zu kritisieren. (R)	I tend to criticize others. (R)	nonmonotonically increasing
BFI_A10	Ich bin nachsichtig, vergebe anderen leicht.	I am indulgent and have a forgiving nature.	inverted-U-shaped
BFI_A11	Ich bin anderen gegenüber misstrauisch. (R)	I am suspicious of others. (R)	nonmonotonically increasing
BFI_A12	Ich schenke anderen leicht Vertrauen, glaube an das Gute im Menschen.	I trust others easily and assume the best about people.	inverted-U-shaped
*Modified items, Agreeableness:*			
mod_A01	Ich leide mit den Problemen anderer sehr stark mit.	I am very strongly affected by other people’s problems.	nonmonotonically decreasing
mod_A02	Ich verbringe viel Zeit damit, mich um die Bedürfnisse anderer zu kümmern.	I spend a lot of time taking care of other people’s needs.	inverted-U-shaped
mod_A03	Ich kann mich gut von den Emotionen anderer distanzieren. (R)	I am good at distancing myself from the emotions of others. (R)	nonmonotonically decreasing
mod_A04	Durch die Probleme anderer lasse ich mich nicht von meinen Zielen abbringen. (R)	I don’t let the problems of others distract me from my goals. (R)	nonmonotonically decreasing
mod_A05	Ich kann nur schwer Entscheidungen treffen, welche andere verletzen könnten.	I find it difficult to make decisions that could hurt others.	nonmonotonically decreasing
mod_A06	Ich kann anderen Personen nur schwer einen Wunsch ausschlagen.	I find it difficult to refuse other people a wish.	nonmonotonically decreasing
mod_A07	Unangenehme Gespräche zu führen, macht mir nichts aus. (R)	I don’t mind having uncomfortable conversations. (R)	monotonically decreasing
mod_A08	Ich scheue mich nicht vor hitzigen Diskussionen. (R)	I don’t shy away from heated discussions. (R)	nonmonotonically decreasing
mod_A09	Ich gebe oft nach, um Streit zu vermeiden.	I often give in to avoid arguments.	nonmonotonically decreasing
mod_A10	Ich gebe lieber nach, als eine Meinungsverschiedenheit auszudiskutieren.	I would rather give in than argue out a difference of opinion.	monotonically decreasing
mod_A11	Ich bin ein sehr harmoniebedürftiger Mensch.	I am a very harmony-seeking person.	inverted-U-shaped
mod_A12	Kritik an anderen zu äußern, fällt mir nicht schwer. (R)	I don’t find it difficult to criticize others. (R)	nonmonotonically decreasing
mod_A13	Ich bin sehr nachsichtig.	I am very indulgent.	inverted-U-shaped
mod_A14	Mir fällt es schwer, auch einmal „nein“ zu sagen.	I find it hard to say “no” once in a while.	monotonically decreasing
*BFI-2 items, Conscientiousness:*			
BFI_C01	Ich bin eher unordentlich. (R)	I am rather messy. (R)	monotonically increasing
BFI_C02	Ich bin systematisch, halte meine Sachen in Ordnung.	I am systematic and keep things in order.	nonmonotonically increasing
BFI_C03	Ich mag es sauber und aufgeräumt.	I like to keep things neat and tidy.	nonmonotonically increasing
BFI_C04	Ich bin eher der chaotische Typ, mache selten sauber. (R)	I am more of a chaotic type and rarely clean up. (R)	monotonically increasing
BFI_C05	Ich bin bequem, neige zu Faulheit. (R)	I tend to be lazy. (R)	monotonically increasing
BFI_C06	Ich neige dazu, Aufgaben vor mir herzuschieben. (R)	I have difficulty getting started on tasks. (R)	monotonically increasing
BFI_C07	Ich bin effizient, erledige Dinge schnell.	I am efficient and get things done quickly.	monotonically increasing
BFI_C08	Ich bleibe an einer Aufgabe dran, bis sie erledigt ist.	I am persistent and work until the task is finished.	monotonically increasing
BFI_C09	Ich bin stetig, beständig.	I am dependable, steady.	nonmonotonically increasing
BFI_C10	Ich bin manchmal ziemlich nachlässig. (R)	I can occasionally be somewhat careless. (R)	monotonically increasing
BFI_C11	Ich bin verlässlich, auf mich kann man zählen.	I am a reliable person one can count on.	monotonically increasing
BFI_C12	Manchmal verhalte ich mich verantwortungslos, leichtsinnig. (R)	I sometimes behave irresponsibly and recklessly. (R)	monotonically increasing
*Modified items, Conscientiousness:*			
mod_C01	Ich verliere viel Zeit damit, meine Sachen zu ordnen.	I lose a lot of time organizing my things.	monotonically decreasing
mod_C02	Beim Thema Ordnung bin ich nicht pingelig. (R)	I am not picky when it comes to tidiness. (R)	nonmonotonically increasing
mod_C03	Ich bin penibel.	I am fastidious.	inverted-U-shaped
mod_C04	Ich bin als Perfektionist bekannt.	I am known as a perfectionist.	inverted-U-shaped
mod_C05	Dem Ordnen von Dokumenten und Dateien räume ich viel Zeit ein.	I spend a lot of time organizing documents and files.	inverted-U-shaped
mod_C06	Von Plänen weiche ich nur ungern ab.	I don’t like to deviate from plans.	inverted-U-shaped
mod_C07	Wenn ich Aufgaben nicht sofort erledigen kann, fühle ich mich schlecht.	If I can’t complete tasks immediately, I feel bad.	inverted-U-shaped
mod_C08	Ich kann unabgeschlossene Projekte auch einmal für einige Zeit ruhen lassen. (R)	I can also let unfinished projects rest for a while. (R)	inverted-U-shaped
mod_C09	Ich verbeiße mich in Aufgaben, bis ich zu einer Lösung gelange.	I get wound up in tasks until I reach a solution.	nonmonotonically increasing
mod_C10	Alltägliche Aufgaben erledige ich so sorgfältig, dass ich oft länger brauche als erforderlich.	I perform everyday tasks so thoroughly that I often need longer than necessary.	nonmonotonically decreasing
mod_C11	Selbst wenn ich einen belanglosen Fehler mache, kann ich diesen nur schwer akzeptieren.	Even if I make a trivial mistake, I find it hard to accept.	monotonically decreasing
mod_C12	Auch bei unwichtigen Projekten arbeite ich sehr akribisch.	I work very meticulously even on unimportant projects.	nonmonotonically increasing
mod_C13	Es macht mir nichts aus, Dinge aufzuschieben. (R)	I don’t mind putting things off. (R)	nonmonotonically increasing
*BFI-2 items, Emotional Stability:*			
BFI_N01	Ich bleibe auch in stressigen Situationen gelassen. (R)	I stay calm even in stressful situations. (R)	monotonically increasing
BFI_N02	Ich reagiere leicht angespannt.	I easily react tensely.	monotonically increasing
BFI_N03	Ich mache mir oft Sorgen.	I worry a lot.	nonmonotonically increasing
BFI_N04	Ich werde selten nervös und unsicher. (R)	I rarely feel anxious and insecure. (R)	monotonically increasing
BFI_N05	Ich bleibe auch bei Rückschlägen zuversichtlich. (R)	I stay confident after experiencing a setback. (R)	monotonically increasing
BFI_N06	Ich bin selbstsicher, mit mir zufrieden. (R)	I am self-confident and content with me. (R)	monotonically increasing
BFI_N07	Ich fühle mich oft bedrückt, freudlos.	I often feel sad, joyless.	monotonically increasing
BFI_N08	Ich bin oft deprimiert, niedergeschlagen.	I tend to feel depressed, blue.	monotonically increasing
BFI_N09	Ich kann launisch sein, habe schwankende Stimmungen.	I can be moody and have up-and-down mood swings.	monotonically increasing
BFI_N10	Ich bin ausgeglichen, nicht leicht aus der Ruhe zu bringen. (R)	I am even-tempered, not easily upset. (R)	monotonically increasing
BFI_N11	Ich habe meine Gefühle unter Kontrolle, werde selten wütend. (R)	I have my emotions under control and rarely get angry. (R)	monotonically increasing
BFI_N12	Ich reagiere schnell gereizt oder genervt.	I quickly become irritated or annoyed.	monotonically increasing
*Modified items, Emotional Stability:*			
mod_N01	Kaum etwas kann meinen emotionalen Zustand verändern. (R)	Hardly anything can change my emotional state. (R)	inverted-U-shaped
mod_N02	Meine Stimmung hängt nicht von äußeren Umständen ab. (R)	My mood does not depend on external circumstances. (R)	monotonically increasing
mod_N03	Auf bedeutsame Ereignisse reagiere ich unemotional. (R)	I react unemotionally to significant events. (R)	nonmonotonically decreasing
mod_N04	Ich bin sensibel für meine Gefühle und Stimmungen.	I am sensitive to my feelings and moods.	inverted-U-shaped
mod_N05	Selbst in gefährlichen Situationen verspüre ich keine Angst. (R)	Even in dangerous situations, I feel no fear. (R)	inverted-U-shaped
mod_N06	Ich erkenne Risiken sehr früh.	I recognize risks very early.	monotonically decreasing
mod_N07	Nichts kann dazu führen, dass ich niedergeschlagen bin. (R)	Nothing can cause me to be dejected. (R)	inverted-U-shaped
mod_N08	Meinungsverschiedenheiten können mich nach Feierabend weiter verfolgen.	Differences of opinion can continue to haunt me after work.	nonmonotonically increasing
mod_N09	Kritik an meiner Arbeit lässt mich kalt. (R)	Criticism of my work leaves me cold. (R)	nonmonotonically decreasing
mod_N10	Persönliche Kritik kann mir nichts anhaben. (R)	Personal criticism cannot harm me. (R)	nonmonotonically increasing
mod_N11	Ich empfinde selten starke Emotionen. (R)	I rarely feel strong emotions. (R)	inverted-U-shaped
mod_N12	Auf der Arbeit ist es noch niemandem gelungen, mich emotional zu verletzen. (R)	No one at work has ever managed to hurt me emotionally. (R)	nonmonotonically increasing
mod_N13	Gelegentlich merke ich, dass ich leicht verletzlich bin.	Occasionally I notice that I am slightly vulnerable.	nonmonotonically increasing
*BFI-2 items, Openness:*			
BFI_O01	Ich bin nicht sonderlich kunstinteressiert. (R)	I am not particularly interested in art. (R)	inverted-U-shaped
BFI_O02	Ich kann mich für Kunst, Musik und Literatur begeistern.	I can be fascinated by art, music, and literature.	inverted-U-shaped
BFI_O03	Ich weiß Kunst und Schönheit zu schätzen.	I value art and beauty.	inverted-U-shaped
BFI_O04	Ich finde Gedichte und Theaterstücke langweilig. (R)	I think poetry and plays are boring. (R)	inverted-U-shaped
BFI_O05	Ich bin vielseitig interessiert.	I have a wide range of interests.	monotonically increasing
BFI_O06	Ich meide philosophische Diskussionen. (R)	I avoid philosophical discussions. (R)	inverted-U-shaped
BFI_O07	Es macht mir Spaß, gründlich über komplexe Dinge nachzudenken und sie zu verstehen.	I enjoy thinking deeply about complex things and understanding them.	monotonically increasing
BFI_O08	Mich interessieren abstrakte Überlegungen wenig. (R)	I have little interest in abstract ideas. (R)	nonmonotonically increasing
BFI_O09	Ich bin erfinderisch, mir fallen raffinierte Lösungen ein.	I am inventive and find clever ways to do things.	monotonically increasing
BFI_O10	Ich bin nicht besonders einfallsreich. (R)	I have little creativity. (R)	monotonically increasing
BFI_O11	Ich bin nicht sonderlich fantasievoll. (R)	I have difficulty imagining things. (R)	nonmonotonically increasing
BFI_O12	Ich bin originell, entwickle neue Ideen.	I am original and come up with new ideas.	monotonically increasing
*Modified items, Openness:*			
mod_O01	Ich kann mich in Kunst, Musik und Literatur verlieren.	I can lose myself in art, music, and literature.	nonmonotonically decreasing
mod_O02	Ich kann Fantasien nicht viel abgewinnen. (R)	I don’t take much pleasure in fantasizing. (R)	nonmonotonically increasing
mod_O03	Ich setze Projekte lieber praktisch um, als mich mit theoretischen Aspekten zu beschäftigen. (R)	I prefer to put projects into practice than deal with theoretical aspects. (R)	inverted-U-shaped
mod_O04	Routinetätigkeiten langweilen mich schnell.	Routine tasks bore me quickly.	nonmonotonically decreasing
mod_O05	Neue Aufgaben ziehe ich Tätigkeiten vor, mit denen ich mich auskenne.	I prefer new tasks to activities that I am familiar with.	inverted-U-shaped
mod_O06	Bei neuen Problemen greife ich auf altbewährte Methoden zurück. (R)	I fall back on tried and tested methods when faced with new problems. (R)	inverted-U-shaped
mod_O07	Ich finde jedes Mal einen neuen Weg, an eine bekannte Aufgabe heranzugehen.	I always find a new approach to a familiar task.	nonmonotonically increasing
mod_O08	Ich verweile nicht lange in Träumen und Fantasien. (R)	I don’t linger in dreams and fantasies for long. (R)	nonmonotonically decreasing
mod_O09	Ich habe oft träumerische Gedanken.	I often have dreamy thoughts.	nonmonotonically decreasing
mod_O10	Meine Herangehensweisen an Aufgaben sind oft unkonventionell.	My approach to tasks is often unconventional.	inverted-U-shaped
mod_O11	Ich bin sehr experimentierfreudig.	I am very keen to experiment.	nonmonotonically increasing
mod_O12	Meine Ideen sind oftmals weit hergeholt.	My ideas are often far-fetched.	nonmonotonically decreasing

*Note.* The classification of desirability trajectories for negatively keyed items is for the case of recoded item responses. BFI-2 = *Big Five Inventory 2* (Danner et al., 2016, 2019); (R) = negatively-keyed items

## Footnotes

1 This effect is illustrated in empirical data in Supplement II.

2 In this article, we refer to the case that items are coded such that higher categories reflect higher substantive trait levels. Also, we refer to the usual case in high-stakes personality assessments that the measured substantive traits are – on a superordinate level (i.e., independent of the content of particular items) – desirable. For substantive traits that are undesirable on a superordinate level, such as aversive personality traits, the logic reverses, that is, lower categories are generally associated with higher desirability levels.

3 We also fitted a model only accounting for substantive traits as well as a model accounting for substantive traits and faking to all datasets. For the sake of simplicity, we provide the simulation results including these two alternative models in Supplement I. Conclusions regarding the simulation factors were identical.

4 Random noise was generated by simulating desirability ratings of *n* = 100 hypothetical pilot study participants for each item in every replication. These ratings were based on the items’ true desirability trajectories and featured a judgment of the most desirable response category for each item from every hypothetical pilot study participant. Faking scoring weights were then derived by transforming the relative frequencies of the simulated ratings per item to a range from 0 to 6.

5 We also ran an ANOVA that additionally featured the repeated-measures factor *Substantive Trait* to allow for systematic differences between the five substantive traits that had been generated to have different latent correlations with faking. The main effect of *Substantive Trait* was ${\eta }_{G}^{2}$ = .083. All interactions including the *Substantive Trait* factor had ${\eta }_{G}^{2}$s < .055. Conclusions regarding the other simulation factors were identical.

6 As an additional analysis, we examined the recovery of person parameters of the faking dimension depending on simulation conditions. Results can be found in Table S.I.2 and Figure S.I.1 in Supplement I. Conclusions regarding the simulation factors were very similar to the ones for the recovery of substantive trait person parameters.

7 As an additional analysis, we examined the recovery of latent correlations between the faking dimension and substantive traits depending on simulation conditions. Results can be found in Table S.I.2 and Figure S.I.2 in Supplement I. Conclusions regarding the simulation factors were very similar to the ones for the recovery of latent correlations between substantive traits.

8 Note that this logic reverses for negatively-keyed items. These items are phrased such that lower categories represent higher levels of the substantive trait. Hence, these items as well as their desirability trajectories need to be recoded.

9 After data collection, we calculated the mean absolute deviation between item responses and modes of desirability ratings from the pilot study for each participant of the main study across all 125 items. The 10% of participants with the smallest mean absolute deviation received the bonus compensation.

10 Peabody (Peabody, 1967, 1984) and other scholars (e.g., Borkenau & Ostendorf, 1989; Petterson et al., 2012; Saucier, 1994) proposed an approach of deconfounding descriptive and evaluative aspects that is similar to our approach. Namely, they created sets of four adjectives that are balanced with respect to the direction of descriptive and the valence of evaluative item content. Their studies, however, were limited to trait inferences from adjectives and did not feature typical personality test items in the form of statements that test-takers can more or less agree with. Also, they did not account for faking in a model-based manner using these kinds of items.

## Availability of code

R scripts for the simulation and the empirical analyses as well as detailed explanations for specifying and estimating the MNRM are available at https://osf.io/ms57p/.

## Declarations

### Preregistration

Analyses in this research were not preregistered because the purpose of the study was the evaluation of a psychometric model through a statistical simulation. The part of the study involving empirical data served as an illustration of the simulation findings in empirical questionnaire data and did not involve a confirmatory testing of hypotheses.
