# Supplementary material for: Using the multidimensional nominal response model to model faking in questionnaire data: The importance of item desirability characteristics
Source: Behav Res Methods. 2024 Sep 20;56(8):8869–96. doi: 10.3758/s13428-024-02509-x (PMC11525249; doi:10.3758/s13428-024-02509-x)
Supplement: Supplementary file 1 — Supplementary file1 (DOCX 1.07 MB) [file 13428_2024_2509_MOESM1_ESM.docx]

# Supplement I

## Effect Size Estimates of Simulation Factors (as Reported in the Main Text)

**Table S.I.1**

*Effect Size Estimates of Simulation Factors*

|  | Recovery of substantive trait person parameters |  | Recovery of latent correlations between substantive traits | |
| --- | --- | --- | --- | --- |
| Effect | Correlation between estimated and true parameters |  | Bias of estimated parameters | RMSE of estimated parameters |
| Mod | .437 |  | .257 | .452 |
| IDC | .565 |  | .567 | .272 |
| TL | .678 |  | .019 | .031 |
| SS | .002 |  | .001 | .025 |
| PRS | .006 |  | .007 | .000 |
| FI | .864 |  | .594 | .733 |
| Mod×IDC | .139 |  | .270 | .057 |
| Mod×TL | .024 |  | .000 | .023 |
| Mod×D | .000 |  | .000 | .002 |
| Mod×PRS | .001 |  | .003 | .000 |
| Mod×FI | .414 |  | .097 | .381 |
| IDC×TL | .039 |  | .018 | .014 |
| IDC×SS | .000 |  | .001 | .002 |
| IDC×PRS | .001 |  | .002 | .005 |
| IDC×FI | .434 |  | .579 | .195 |
| TL×SS | .000 |  | .001 | .002 |
| TL×PRS | .000 |  | .001 | .003 |
| TL×FI | .132 |  | .000 | .026 |
| SS×PRS | .000 |  | .000 | .000 |
| SS×FI | .000 |  | .001 | .002 |
| PRS×FI | .004 |  | .000 | .001 |
| Mod×IDC×TL | .013 |  | .009 | .015 |
| Mod×IDC×SS | .000 |  | .001 | .000 |
| Mod×IDC×PRS | .003 |  | .001 | .005 |
| Mod×IDC×FI | .204 |  | .296 | .165 |
| Mod×TL×SS | .000 |  | .001 | .001 |
| Mod×TL×PRS | .001 |  | .000 | .001 |
| Mod×TL×FI | .022 |  | .004 | .007 |
| Mod×SS×PRS | .000 |  | .000 | .000 |
| Mod×SS×FI | .000 |  | .000 | .001 |
| Mod×PRS×FI | .000 |  | .001 | .000 |
| IDC×TL×SS | .000 |  | .001 | .000 |
| IDC×TL×PRS | .001 |  | .001 | .001 |
| IDC×TL×FI | .040 |  | .025 | .030 |
| IDC×SS×PRS | .000 |  | .003 | .000 |
| IDC×SS×FI | .001 |  | .001 | .001 |
| IDC×PRS×FI | .003 |  | .001 | .006 |
| TL×SS×PRS | .000 |  | .000 | .000 |
| TL×SS×FI | .000 |  | .000 | .000 |
| TL×PRS×FI | .000 |  | .000 | .000 |
| SS×PRS×FI | .000 |  | .000 | .000 |
| Mod×IDC×TL×SS | .000 |  | .000 | .000 |
| Mod×IDC×TL×PRS | .000 |  | .001 | .001 |
| Mod×IDC×TL×FI | .030 |  | .011 | .037 |
| Mod×IDC×SS×PRS | .000 |  | .001 | .000 |
| Mod×IDC×SS×FI | .000 |  | .001 | .001 |
| Mod×IDC×PRS×FI | .005 |  | .000 | .007 |
| Mod×TL×SS×PRS | .000 |  | .000 | .000 |
| Mod×TL×SS×FI | .000 |  | .000 | .000 |
| Mod×TL×PRS×FI | .000 |  | .000 | .000 |
| Mod×SS×PRS×FI | .000 |  | .000 | .000 |
| IDC×TL×SS×PRS | .000 |  | .001 | .000 |
| IDC×TL×SS×FI | .001 |  | .001 | .000 |
| IDC×TL×PRS×FI | .002 |  | .001 | .002 |
| IDC×SS×PRS×FI | .001 |  | .002 | .000 |
| TL×SS×PRS×FI | .000 |  | .000 | .000 |
| Mod×IDC×TL×SS×PRS | .000 |  | .001 | .000 |
| Mod×IDC×TL×SS×FI | .000 |  | .001 | .001 |
| Mod×IDC×TL×PRS×FI | .001 |  | .002 | .002 |
| Mod×IDC×SS×PRS×FI | .000 |  | .002 | .001 |
| Mod×TL×SS×PRS×FI | .000 |  | .000 | .000 |
| IDC×TL×SS×PRS×FI | .001 |  | .001 | .001 |
| Mod×IDC×TL×SS×PRS×FI | .000 |  | .001 | .001 |
|  |  |  |  |  |

*Note.* Labeling of simulation factors: Mod = *Model*; IDC = *Item Desirability Characteristics*; TL = *Test Length*; SS = *Sample Size*; PRS = *Presence of Response Styles*; FI = *Faking Impact*. Effect size estimates are generalized *η*^2^ values ($\text{η}_{\text{G}}^{\text{2}}$; cf. Olejnik & Algina, 2003). Correlations between estimated and true substantive traits person parameters were Fisher-*z*-transformed. RMSE = root mean square error.

## Additional Simulation Results

### Recovery of Faking Person Parameters and Latent Correlations Between Faking and Substantive Traits

Parameter recovery of person parameters of the faking dimension as well as latent correlations between faking and substantive traits is reported in the following. Table S.I.2 contains effect size estimates of the simulation factors. Figures S.I.1 and S.I.2 depict parameter recovery graphically.

**Table S.I.2**

*Effect Size Estimates of Simulation Factors*

|  | Recovery of faking person parameters |  | Recovery of latent correlations between faking and substantive traits | |
| --- | --- | --- | --- | --- |
| Effect | Correlation between estimated and true parameters |  | Bias of estimated parameters | RMSE of estimated parameters |
| IDC | .810 |  | .527 | .677 |
| TL | .537 |  | .025 | .024 |
| SS | .012 |  | .000 | .009 |
| PRS | .027 |  | .001 | .002 |
| FI | .988 |  | .540 | .554 |
| IDC×TL | .006 |  | .028 | .043 |
| IDC×SS | .007 |  | .001 | .001 |
| IDC×PRS | .013 |  | .001 | .001 |
| IDC×FI | .694 |  | .462 | .597 |
| TL×SS | .000 |  | .000 | .000 |
| TL×PRS | .000 |  | .001 | .000 |
| TL×FI | .384 |  | .002 | .025 |
| SS×PRS | .001 |  | .001 | .001 |
| SS×FI | .008 |  | .001 | .000 |
| PRS×FI | .022 |  | .000 | .003 |
| IDC×TL×SS | .001 |  | .001 | .001 |
| IDC×TL×PRS | .000 |  | .000 | .000 |
| IDC×TL×FI | .003 |  | .007 | .029 |
| IDC×SS×PRS | .001 |  | .001 | .001 |
| IDC×SS×FI | .003 |  | .001 | .002 |
| IDC×PRS×FI | .006 |  | .001 | .001 |
| TL×SS×PRS | .000 |  | .000 | .000 |
| TL×SS×FI | .000 |  | .001 | .000 |
| TL×PRS×FI | .000 |  | .003 | .001 |
| SS×PRS×FI | .001 |  | .000 | .001 |
| IDC×TL×SS×PRS | .001 |  | .000 | .000 |
| IDC×TL×SS×FI | .001 |  | .001 | .001 |
| IDC×TL×PRS×FI | .000 |  | .001 | .001 |
| IDC×SS×PRS×FI | .001 |  | .001 | .001 |
| TL×SS×PRS×FI | .000 |  | .000 | .000 |
| IDC×TL×SS×PRS×FI | .002 |  | .001 | .001 |
|  |  |  |  |  |

*Note.* Labeling of simulation factors: IDC = *Item Desirability Characteristics*; TL = *Test Length*; SS = *Sample Size*; PRS = *Presence of Response Styles*; FI = *Faking Impact*. Effect size estimates are generalized *η*^2^ values ($\text{η}_{\text{G}}^{\text{2}}$; cf. Olejnik & Algina, 2003). Correlations between estimated and true faking person parameters were Fisher-*z*-transformed. RMSE = root mean square error.

**Figure S.I.1**

*Recovery of Faking Person Parameters*


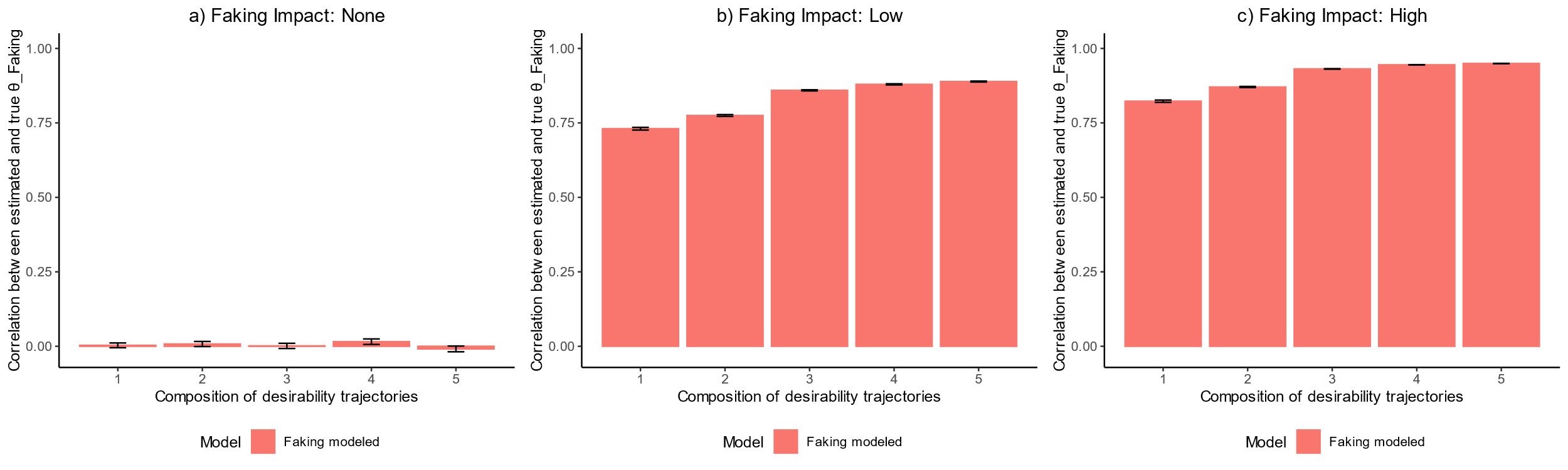


*Note.* The depicted recovery of faking person parameters is for the representative case of 6 items per substantive trait scale, a sample size of 1000, and extreme response style (ERS) being present in the data. Models accounting for faking included dimensions for substantive traits, ERS, and faking. Values reflect the back-transformed mean of the Fisher-*z*-transformed correlations between estimated and true person parameters across replications within a condition. Error bars represent the standard error of the mean.

**Figure S.I.2**

*Recovery of Latent Correlations Between Faking and Substantive Traits*


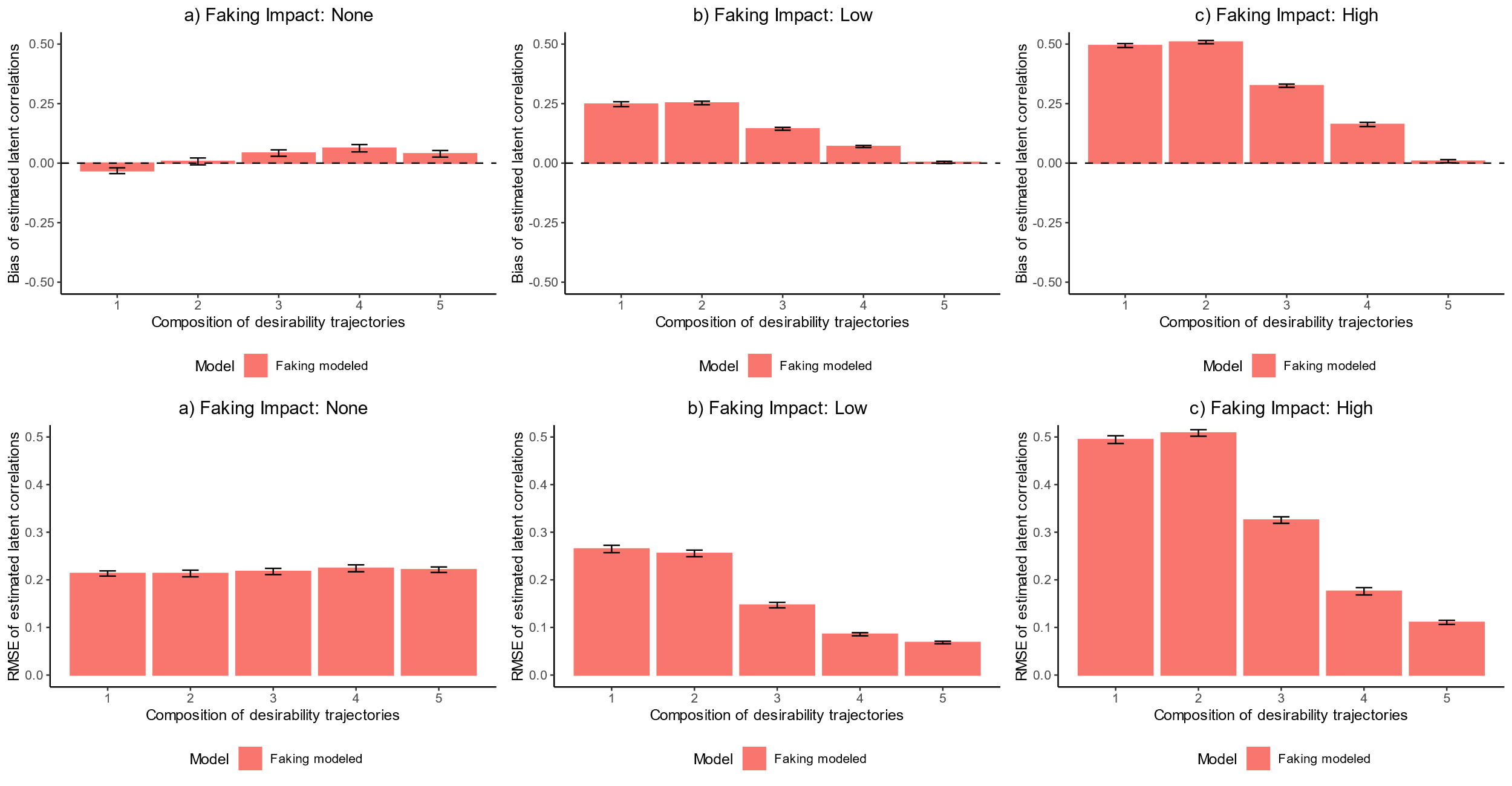


*Note.* The depicted recovery of latent correlations between faking and substantive traits is for the representative case of 6 items per substantive trait scale, a sample size of 1000, and extreme response style (ERS) being present in the data. Models accounting for faking included dimensions for substantive traits, ERS, and faking. Values reflect the mean bias (upper panel) and root mean square error (RMSE; lower panel) across replications within a condition. Error bars represent the standard error of the mean.

### Parameter Recovery in Comparison to Alternative Models

In the following, recovery of the considered model parameters is depicted in comparison to parameter recovery in alternative models (Figures S.I.3 to S.I.6), namely in a model only accounting for substantive traits and in a model accounting for substantive traits and faking.

**Figure S.I.3**

*Recovery of Substantive Trait Person Parameters*


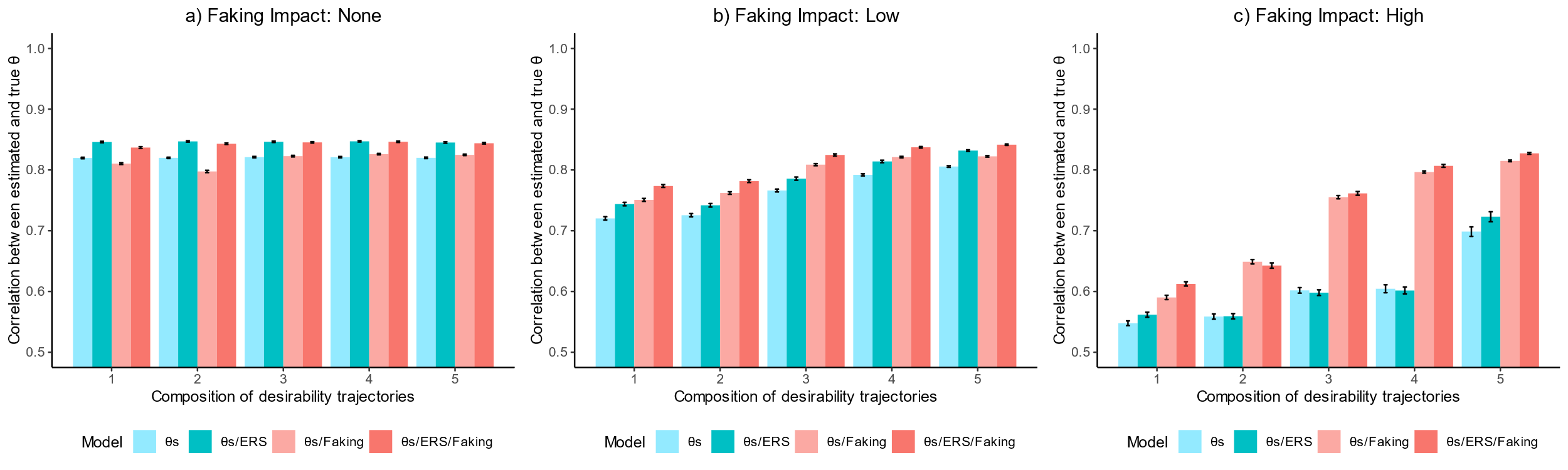


*Note.* The depicted recovery of substantive trait person parameters is for the representative case of 6 items per substantive trait scale, a sample size of 1000, and extreme response style (ERS) being present in the data. θs = only substantive traits modeled; θs/ERS = substantive traits and ERS modeled; θs/Faking = substantive traits and faking modeled; θs/ERS/Faking = substantive traits, ERS, and faking modeled. Results are aggregated across the five substantive traits used in the simulation. Values reflect the back-transformed mean of the Fisher-*z*-transformed correlations between estimated and true person parameters across replications within a condition. Error bars represent the standard error of the mean.

**Figure S.I.4**

*Recovery of Latent Correlations Between Substantive Traits*


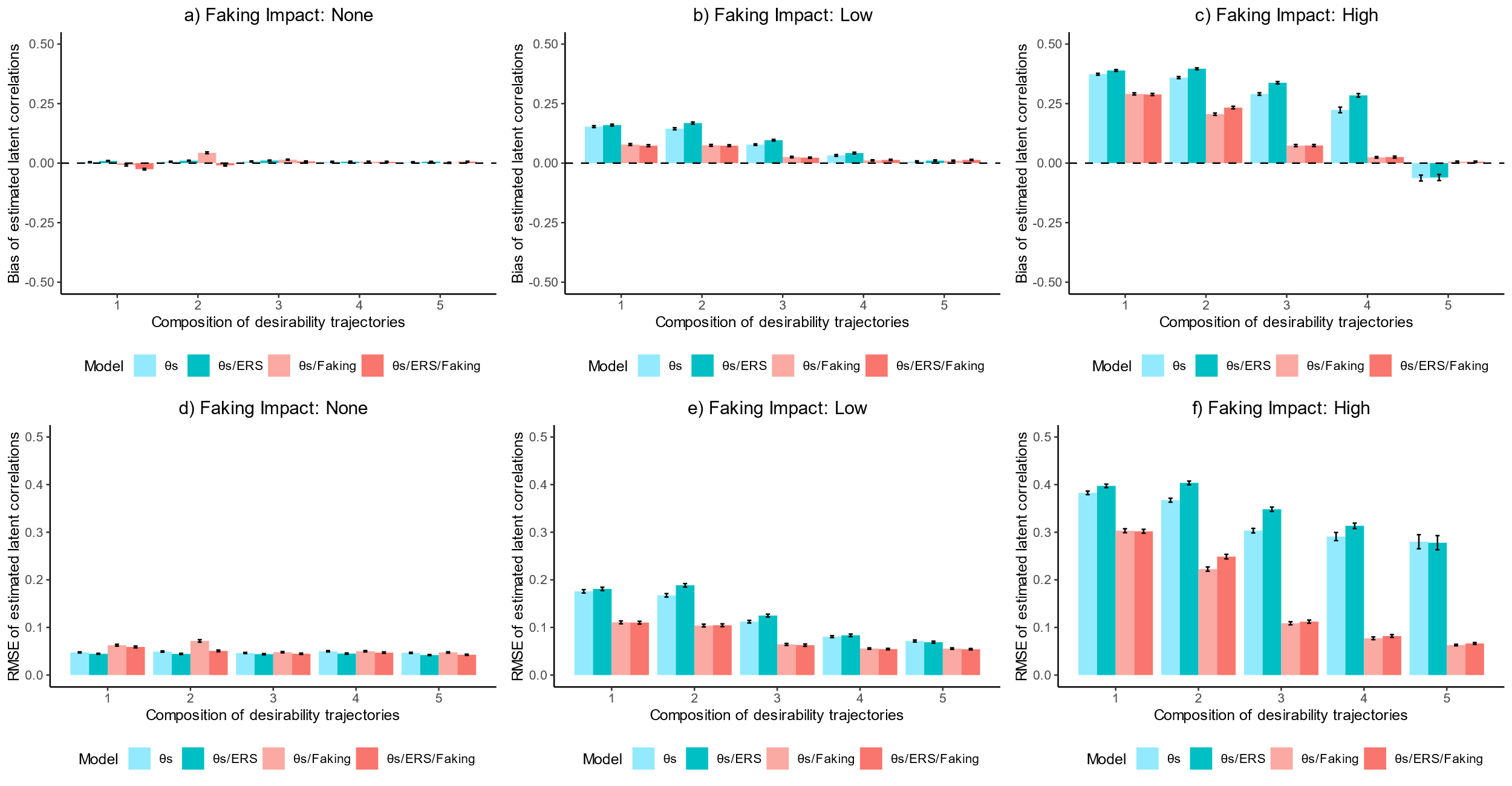


*Note.* The depicted recovery of latent correlations between substantive traits is for the representative case of 6 items per substantive trait scale, a sample size of 1000, and extreme response style (ERS) being present in the data. θs = only substantive traits modeled; θs/ERS = substantive traits and ERS modeled; θs/Faking = substantive traits and faking modeled; θs/ERS/Faking = substantive traits, ERS, and faking modeled. Values reflect the mean bias (upper panel) and root mean square error (RMSE; lower panel) across replications within a condition. Error bars represent the standard error of the mean.

**Figure S.I.5**

*Recovery of Faking Person Parameters*


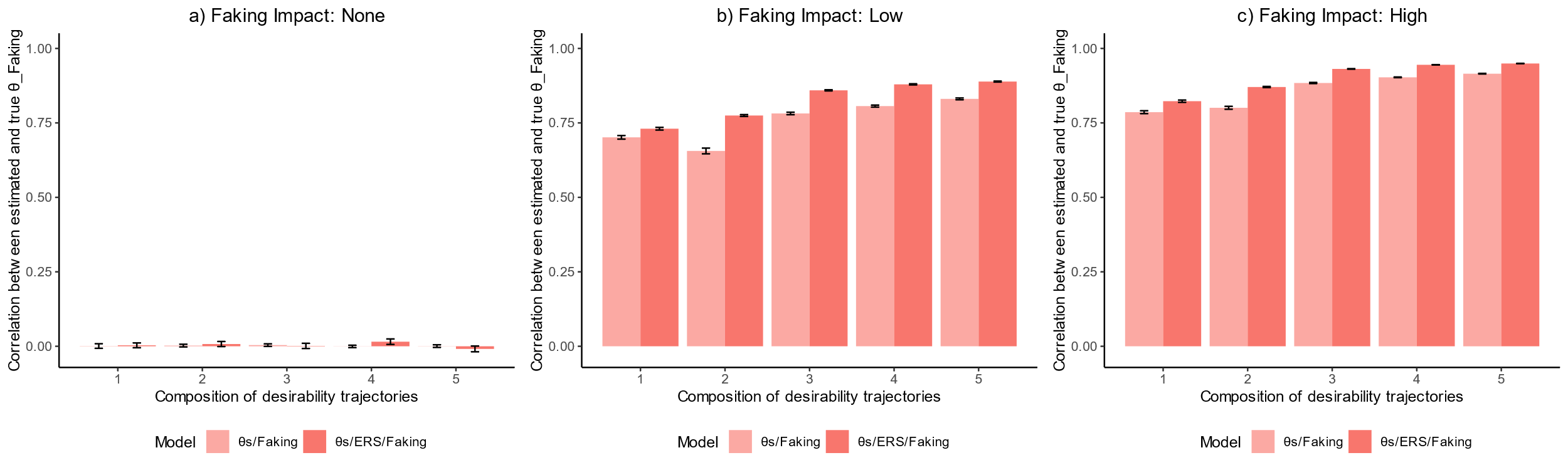


*Note.* The depicted recovery of faking person parameters is for the representative case of 6 items per substantive trait scale, a sample size of 1000, and extreme response style (ERS) being present in the data. θs/Faking = substantive traits and faking modeled; θs/ERS/Faking = substantive traits, ERS, and faking modeled. Values reflect the back-transformed mean of the Fisher-*z*-transformed correlations between estimated and true person parameters across replications within a condition. Error bars represent the standard error of the mean.

**Figure S.I.6**

*Recovery of Latent Correlations Between Faking and Substantive Traits*


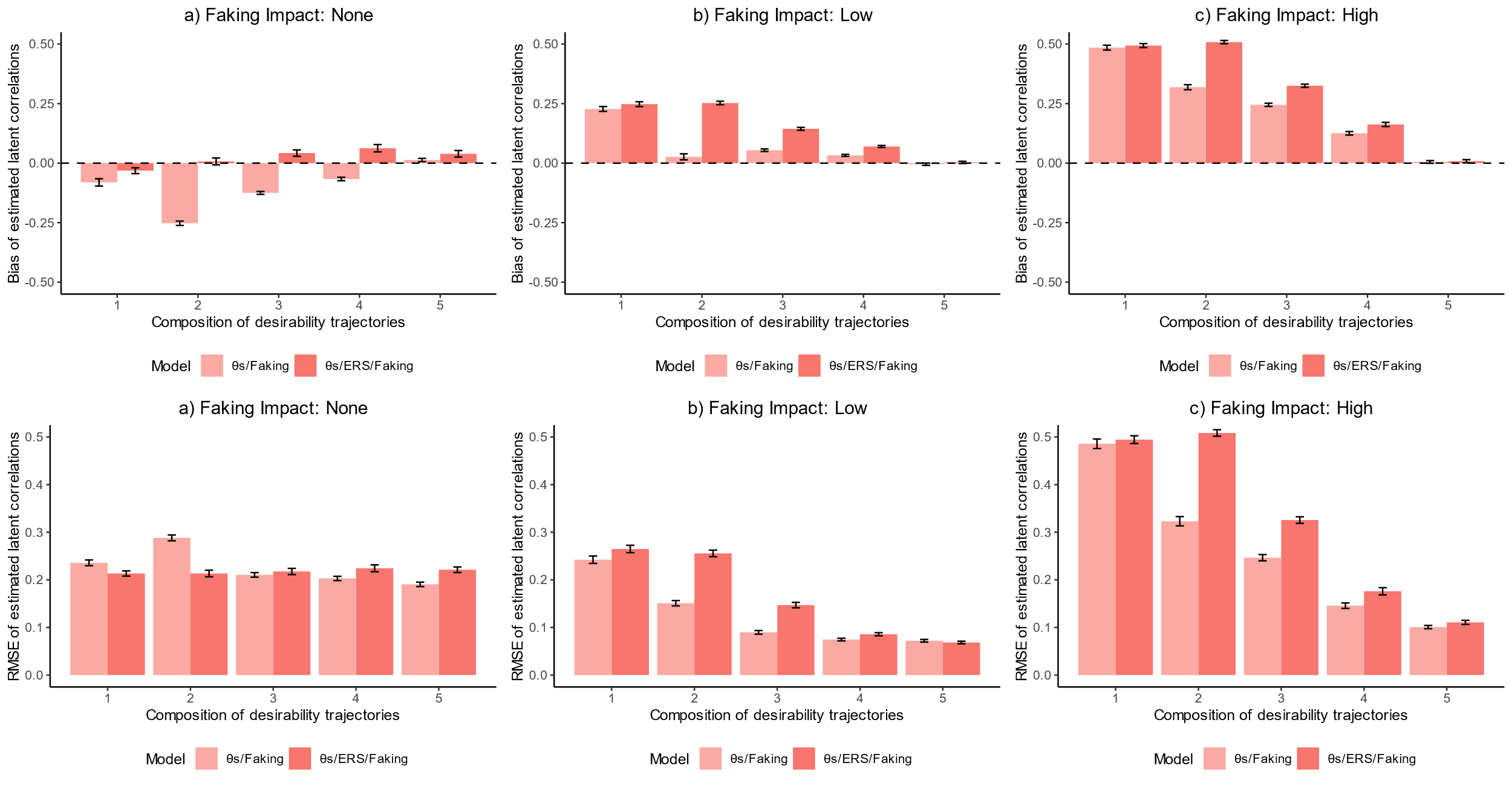


*Note.* The depicted recovery of latent correlations between faking and substantive traits is for the representative case of 6 items per substantive trait scale, a sample size of 1000, and extreme response style (ERS) being present in the data. θs/Faking = substantive traits and faking modeled; θs/ERS/Faking = substantive traits, ERS, and faking modeled. Results are aggregated across the five substantive traits used in the simulation. Values reflect the mean bias (upper panel) and root mean square error (RMSE; lower panel) across replications within a condition. Error bars represent the standard error of the mean.

# References

Olejnik, S., & Algina, J. (2003). Generalized eta and omega squared statistics: Measures of effect size for some common research designs. *Psychological Methods, 8*(4), 434–447. [https://doi.org/1.1037/1082-989X.8.4.434](https://doi.org/10.1037/1082-989X.8.4.434)
