# Supplementary material for: Using the multidimensional nominal response model to model faking in questionnaire data: The importance of item desirability characteristics
Source: Behav Res Methods. 2024 Sep 20;56(8):8869–96. doi: 10.3758/s13428-024-02509-x (PMC11525249; doi:10.3758/s13428-024-02509-x)
Supplement: Supplementary file 2 — Supplementary file2 (DOCX 106 KB) [file 13428_2024_2509_MOESM2_ESM.docx]

# Supplement II

## Item Modification Strategies

When modifying the items from the German version of the *Big Five Inventory 2* (BFI-2; Danner et al., 2016, 2019), we followed certain strategies to create more variety in desirability trajectories with respect to the social situation of an application for a leadership position in the industry. At the same time, we always considered the construct definitions of the Big Five (McCrae & Costa, 1987) in order to retain the core of the traits of interest. The item modification strategies were the following:

1. Item rewording based on the conducted job demand analysis
   - According to the survey of persons currently holding a leadership position, we derived attitudes, behaviors, and attributes that were regarded as not ideal in the specific context (experiential-intuitive approach; see Schuler, 2014; Höft, 2019) but at the same time could be assumed as manifestations of one of the Big Five traits.
   - According to the leadership literature (see references in the Main Text), we derived attitudes, behaviors, and attributes reflecting too high manifestations of a generally desirable Big Five trait.
2. Item rewording based on the approach by Cao et al. (2015)
   - We specified/adapted frequencies or intensities (e.g., addition of an adverb of frequency or change of intensity level that might change social desirability).
   - We specified/adapted conditions (e.g., addition of a conditional sentence that puts an actually desirable attitude/behavior/attribute in an unfavorable light).
3. Item rewording based on dictionary/thesaurus search
   - We looked for synonyms/antonyms of adjectives that carry a different connotation or social desirability.
   - We looked for alternative action intentions that carry a different connotation or social desirability.

## Faking Scoring Weights and Inclusion in Item Compositions

The following table contains the faking scoring weights for both the BFI-2 and modified items from the empirical demonstration. These were based on the relative frequencies of desirability ratings from the pilot study and were linearly transformed to a range from 0 to 6. Also, the table contains information on which items were included in the respective item composition of desirability trajectories. If item proportions per substantive trait scale were not integer or if no more items with a certain desirability trajectory were available, we filled up the item sets with items of neighboring desirability trajectories to ensure a symmetrical distribution of desirability trajectories.

**Table S.II.1**

*Faking Scoring Weights and Inclusion in Item Compositions of Desirability Trajectories for all Items of the Empirical Demonstration*

|  | Faking scoring weights of response category | | | | | | |  | Inclusion in item composition | | | | |
| --- | --- | --- | --- | --- | --- | --- | --- | --- | --- | --- | --- | --- | --- |
| Item code | 0 | 1 | 2 | 3 | 4 | 5 | 6 |  | 1 | 2 | 3 | 4 | 5 |
| *BFI-2 items, Extraversion:* | | |  |  |  |  |  |  |  |  |  |  |  |
| BFI_E01 | 0.12 | 0.23 | 0.35 | 2.54 | 6.00 | 6.00 | 2.31 |  |  | x | x | x | x |
| BFI_E02 | 0.00 | 0.26 | 0.26 | 0.53 | 1.94 | 6.00 | 4.41 |  | x |  |  |  |  |
| BFI_E03 | 1.30 | 3.00 | 2.87 | 6.00 | 3.13 | 2.74 | 0.78 |  |  |  |  |  |  |
| BFI_E04 | 0.11 | 0.57 | 1.70 | 3.28 | 6.00 | 3.74 | 1.81 |  |  |  |  |  |  |
| BFI_E05 | 0.00 | 0.00 | 0.50 | 0.75 | 2.42 | 6.00 | 3.00 |  | x | x | x |  |  |
| BFI_E06 | 0.00 | 0.21 | 0.72 | 0.62 | 2.59 | 5.59 | 6.00 |  | x | x | x | x | x |
| BFI_E07 | 0.00 | 0.00 | 0.38 | 1.41 | 4.03 | 6.00 | 2.44 |  |  |  |  |  |  |
| BFI_E08 | 0.00 | 0.00 | 0.49 | 1.18 | 3.05 | 6.00 | 4.23 |  | x |  |  |  |  |
| BFI_E09 | 0.00 | 0.63 | 1.13 | 5.25 | 6.00 | 5.13 | 0.88 |  |  |  |  |  |  |
| BFI_E10 | 0.00 | 0.00 | 0.79 | 1.70 | 5.43 | 6.00 | 3.28 |  |  |  |  |  |  |
| BFI_E11 | 0.09 | 0.09 | 0.00 | 1.20 | 2.95 | 6.00 | 3.69 |  | x |  |  |  |  |
| BFI_E12 | 0.00 | 0.00 | 0.00 | 0.80 | 3.30 | 6.00 | 5.10 |  | x | x |  |  |  |
| *Modified items, Extraversion:* | | |  |  |  |  |  |  |  |  |  |  |  |
| mod_E01 | 0.53 | 2.11 | 2.84 | 6.00 | 3.47 | 0.95 | 0.11 |  |  |  |  |  |  |
| mod_E02 | 1.22 | 3.33 | 6.00 | 4.00 | 1.44 | 0.78 | 0.11 |  |  |  |  |  |  |
| mod_E03 | 1.57 | 6.00 | 5.35 | 4.70 | 1.70 | 0.52 | 0.00 |  |  |  |  | x | x |
| mod_E04 | 3.57 | 6.00 | 5.14 | 4.14 | 1.71 | 1.00 | 0.14 |  |  |  |  | x |  |
| mod_E05 | 6.00 | 5.76 | 3.60 | 1.80 | 0.48 | 0.48 | 0.12 |  |  |  |  |  | x |
| mod_E06 | 0.00 | 0.95 | 1.36 | 4.50 | 5.05 | 6.00 | 2.86 |  |  | x |  |  |  |
| mod_E07 | 0.61 | 2.08 | 4.04 | 6.00 | 3.92 | 1.71 | 0.24 |  |  |  | x |  | x |
| mod_E08 | 0.56 | 0.89 | 3.11 | 4.11 | 5.56 | 6.00 | 1.56 |  |  | x | x | x |  |
| mod_E09 | 0.26 | 1.79 | 2.68 | 6.00 | 6.00 | 2.04 | 0.64 |  |  |  | x | x | x |
| mod_E10 | 0.32 | 0.63 | 1.68 | 3.89 | 6.00 | 5.05 | 3.05 |  |  |  |  |  |  |
| mod_E11 | 0.37 | 0.86 | 2.20 | 5.27 | 6.00 | 2.82 | 1.10 |  |  |  |  |  |  |
| mod_E12 | 0.67 | 3.22 | 6.00 | 5.89 | 3.89 | 1.78 | 0.33 |  |  |  |  |  |  |
| mod_E13 | 0.35 | 3.18 | 6.00 | 5.76 | 5.76 | 1.76 | 0.24 |  |  |  |  |  |  |
|  |  | reliability estimates of item compositions: | | | | Cronbach’s *α* | |  | .84 | .84 | .86 | .79 | .81 |
|  |  |  |  |  |  | McDonald’s *ω* | |  | .85 | .85 | .86 | .79 | .81 |
|  |  |  | convergent validities of item compositions with the BFI-2: | | | | |  | .94 | .90 | .86 | .80 | .75 |
| *BFI-2 items, Agreeableness:* | | |  |  |  |  |  |  |  |  |  |  |  |
| BFI_A01 | 0.00 | 0.11 | 0.87 | 1.85 | 6.00 | 5.45 | 2.29 |  |  | x | x | x | x |
| BFI_A02 | 0.00 | 0.00 | 0.59 | 0.98 | 3.44 | 6.00 | 3.93 |  | x | x | x | x | x |
| BFI_A03 | 0.00 | 0.98 | 0.73 | 3.67 | 5.39 | 6.00 | 1.84 |  |  | x | x | x |  |
| BFI_A04 | 0.00 | 0.00 | 0.34 | 0.60 | 1.54 | 4.54 | 6.00 |  | x |  |  |  |  |
| BFI_A05 | 0.00 | 0.00 | 0.11 | 0.00 | 0.21 | 1.75 | 6.00 |  | x | x | x |  |  |
| BFI_A06 | 0.00 | 0.00 | 0.00 | 0.64 | 1.73 | 5.45 | 6.00 |  | x |  |  |  |  |
| BFI_A07 | 0.18 | 0.00 | 0.09 | 0.81 | 2.33 | 4.21 | 6.00 |  | x |  |  |  |  |
| BFI_A08 | 0.00 | 0.00 | 0.09 | 0.99 | 1.88 | 4.66 | 6.00 |  | x | x |  |  |  |
| BFI_A09 | 0.14 | 0.55 | 1.91 | 5.73 | 6.00 | 5.45 | 0.95 |  |  |  |  |  |  |
| BFI_A10 | 0.12 | 0.73 | 3.92 | 6.00 | 5.39 | 2.33 | 0.12 |  |  |  |  |  |  |
| BFI_A11 | 0.14 | 0.14 | 0.95 | 6.00 | 6.00 | 5.59 | 1.91 |  |  | x |  |  |  |
| BFI_A12 | 0.24 | 0.73 | 3.43 | 6.00 | 4.78 | 2.82 | 0.61 |  |  |  |  |  |  |
| *Modified items, Agreeableness:* | | |  |  |  |  |  |  |  |  |  |  |  |
| mod_A01 | 1.82 | 6.00 | 5.57 | 4.93 | 2.14 | 0.32 | 0.21 |  |  |  |  | x |  |
| mod_A02 | 0.26 | 1.53 | 4.34 | 5.23 | 6.00 | 1.66 | 0.38 |  |  |  | x | x | x |
| mod_A03 | 1.88 | 3.00 | 6.00 | 4.50 | 2.13 | 1.13 | 0.38 |  |  |  |  |  |  |
| mod_A04 | 3.96 | 6.00 | 5.55 | 3.28 | 1.92 | 1.25 | 0.23 |  |  |  |  |  |  |
| mod_A05 | 2.67 | 6.00 | 3.22 | 2.22 | 1.78 | 0.89 | 0.11 |  |  |  |  |  |  |
| mod_A06 | 1.73 | 6.00 | 4.68 | 1.73 | 0.81 | 0.41 | 0.10 |  |  |  |  | x | x |
| mod_A07 | 5.65 | 6.00 | 3.58 | 0.92 | 0.69 | 0.69 | 0.00 |  |  |  |  |  |  |
| mod_A08 | 2.88 | 6.00 | 5.04 | 1.92 | 1.32 | 0.96 | 0.12 |  |  |  |  |  |  |
| mod_A09 | 1.94 | 6.00 | 4.06 | 1.74 | 0.68 | 0.29 | 0.00 |  |  |  |  |  |  |
| mod_A10 | 3.96 | 6.00 | 3.43 | 1.29 | 1.18 | 0.43 | 0.00 |  |  |  |  |  |  |
| mod_A11 | 0.00 | 2.13 | 4.38 | 6.00 | 3.25 | 1.88 | 1.38 |  |  |  | x |  | x |
| mod_A12 | 2.06 | 6.00 | 4.80 | 2.49 | 0.60 | 0.60 | 0.26 |  |  |  |  |  |  |
| mod_A13 | 0.44 | 2.63 | 5.71 | 6.00 | 4.68 | 2.34 | 0.44 |  |  |  |  |  |  |
| mod_A14 | 4.78 | 6.00 | 2.64 | 0.81 | 0.71 | 0.51 | 0.00 |  |  |  |  |  | x |
|  |  | reliability estimates of item compositions: | | | | Cronbach’s *α* | |  | .74 | .73 | .76 | .78 | .76 |
|  |  |  |  |  |  | McDonald’s *ω* | |  | .76 | .74 | .77 | .79 | .76 |
|  |  |  | convergent validities of item compositions with the BFI-2: | | | | |  | .87 | .92 | .79 | .71 | .65 |
| *BFI-2 items, Conscientiousness:* | | |  |  |  |  |  |  |  |  |  |  |  |
| BFI_C01 | 0.00 | 0.38 | 0.19 | 1.13 | 2.63 | 6.00 | 3.94 |  | x | x | x |  |  |
| BFI_C02 | 0.00 | 0.18 | 0.18 | 1.15 | 3.44 | 6.00 | 2.47 |  |  | x | x | x | x |
| BFI_C03 | 0.00 | 0.75 | 1.25 | 2.88 | 5.75 | 6.00 | 2.38 |  |  | x | x | x |  |
| BFI_C04 | 0.10 | 0.10 | 0.60 | 0.60 | 2.10 | 6.00 | 5.70 |  | x | x | x | x | x |
| BFI_C05 | 0.00 | 0.13 | 0.13 | 0.39 | 0.59 | 2.67 | 6.00 |  |  |  |  |  |  |
| BFI_C06 | 0.00 | 0.09 | 0.64 | 0.27 | 1.55 | 6.00 | 5.27 |  | x |  |  |  |  |
| BFI_C07 | 0.00 | 0.00 | 0.17 | 0.25 | 1.58 | 4.67 | 6.00 |  | x |  |  |  |  |
| BFI_C08 | 0.00 | 0.10 | 0.39 | 0.58 | 3.77 | 6.00 | 3.87 |  | x | x |  |  |  |
| BFI_C09 | 0.00 | 0.20 | 0.10 | 1.93 | 4.47 | 6.00 | 2.75 |  |  |  |  |  |  |
| BFI_C10 | 0.00 | 0.00 | 0.18 | 0.91 | 2.27 | 4.45 | 6.00 |  | x |  |  |  |  |
| BFI_C11 | 0.00 | 0.00 | 0.00 | 0.00 | 0.28 | 2.17 | 6.00 |  |  |  |  |  |  |
| BFI_C12 | 0.00 | 0.07 | 0.00 | 0.20 | 0.80 | 3.07 | 6.00 |  |  |  |  |  |  |
| *Modified items, Conscientiousness:* | | |  |  |  |  |  |  |  |  |  |  |  |
| mod_C01 | 4.00 | 6.00 | 4.22 | 1.33 | 1.22 | 0.00 | 0.11 |  |  |  |  |  |  |
| mod_C02 | 0.18 | 1.29 | 1.66 | 3.78 | 6.00 | 4.43 | 0.74 |  |  |  |  |  |  |
| mod_C03 | 1.13 | 3.50 | 4.38 | 6.00 | 2.75 | 1.00 | 0.25 |  |  |  |  |  |  |
| mod_C04 | 0.51 | 1.46 | 2.06 | 3.77 | 6.00 | 2.06 | 0.94 |  |  |  | x | x | x |
| mod_C05 | 0.83 | 2.90 | 5.07 | 6.00 | 3.10 | 1.97 | 0.41 |  |  |  |  |  |  |
| mod_C06 | 0.67 | 3.87 | 5.33 | 6.00 | 3.20 | 0.93 | 0.27 |  |  |  |  |  |  |
| mod_C07 | 1.27 | 3.12 | 4.85 | 6.00 | 4.38 | 2.31 | 0.69 |  |  |  |  |  |  |
| mod_C08 | 0.36 | 1.20 | 3.84 | 6.00 | 3.72 | 2.88 | 0.24 |  |  |  | x | x | x |
| mod_C09 | 0.40 | 0.93 | 2.80 | 4.80 | 6.00 | 3.60 | 1.73 |  |  |  |  |  |  |
| mod_C10 | 1.80 | 5.64 | 6.00 | 2.88 | 1.32 | 0.60 | 0.00 |  |  |  |  | x | x |
| mod_C11 | 3.20 | 6.00 | 3.36 | 1.68 | 0.88 | 0.32 | 0.24 |  |  |  |  |  | x |
| mod_C12 | 0.71 | 2.86 | 4.86 | 5.14 | 5.71 | 6.00 | 2.71 |  |  | x |  |  |  |
| mod_C13 | 0.21 | 0.32 | 1.18 | 2.25 | 4.29 | 6.00 | 2.04 |  |  |  |  |  |  |
|  |  | reliability estimates of item compositions: | | | | Cronbach’s *α* | |  | .84 | .83 | .80 | .71 | .63 |
|  |  |  |  |  |  | McDonald’s *ω* | |  | .85 | .84 | .81 | .71 | .60 |
|  |  |  | convergent validities of item compositions with the BFI-2: | | | | |  | .97 | .91 | .85 | .76 | .63 |
| *BFI-2 items, Emotional Stability:* | | |  |  |  |  |  |  |  |  |  |  |  |
| BFI_N01 | 0.00 | 0.16 | 0.32 | 0.55 | 1.26 | 3.71 | 6.00 |  | x | x | x |  |  |
| BFI_N02 | 0.00 | 0.21 | 0.54 | 1.07 | 4.50 | 6.00 | 3.96 |  |  |  |  |  |  |
| BFI_N03 | 0.19 | 0.66 | 0.84 | 1.59 | 3.75 | 6.00 | 1.22 |  |  | x | x | x | x |
| BFI_N04 | 0.59 | 1.41 | 1.06 | 0.82 | 2.94 | 6.00 | 5.06 |  |  |  |  |  |  |
| BFI_N05 | 0.21 | 0.32 | 0.11 | 0.96 | 3.00 | 5.68 | 6.00 |  | x |  |  |  |  |
| BFI_N06 | 0.11 | 0.11 | 0.42 | 1.16 | 2.63 | 6.00 | 5.58 |  | x | x |  |  |  |
| BFI_N07 | 0.00 | 0.17 | 0.25 | 0.42 | 1.17 | 4.67 | 6.00 |  | x |  |  |  |  |
| BFI_N08 | 0.00 | 0.00 | 0.29 | 0.43 | 0.87 | 3.40 | 6.00 |  | x |  |  |  |  |
| BFI_N09 | 0.19 | 0.38 | 0.19 | 0.28 | 1.69 | 6.00 | 5.53 |  |  |  |  |  |  |
| BFI_N10 | 0.10 | 0.29 | 0.77 | 0.77 | 1.26 | 5.52 | 6.00 |  | x | x | x | x | x |
| BFI_N11 | 0.00 | 0.29 | 0.19 | 0.95 | 2.00 | 6.00 | 5.05 |  |  |  |  |  |  |
| BFI_N12 | 0.00 | 0.18 | 0.09 | 0.53 | 1.41 | 5.21 | 6.00 |  |  |  |  |  |  |
| *Modified items, Emotional Stability:* | | |  |  |  |  |  |  |  |  |  |  |  |
| mod_N01 | 0.68 | 2.26 | 3.74 | 6.00 | 4.53 | 4.19 | 0.79 |  |  |  |  |  |  |
| mod_N02 | 0.24 | 0.64 | 0.48 | 1.92 | 3.12 | 6.00 | 3.28 |  |  |  |  |  |  |
| mod_N03 | 1.73 | 6.00 | 4.27 | 3.23 | 1.38 | 0.81 | 0.12 |  |  |  |  | x | x |
| mod_N04 | 1.07 | 3.60 | 6.00 | 4.53 | 4.93 | 4.53 | 1.47 |  |  |  |  |  |  |
| mod_N05 | 1.31 | 4.50 | 5.25 | 5.81 | 6.00 | 4.31 | 1.31 |  |  |  | x |  | x |
| mod_N06 | 5.31 | 6.00 | 3.15 | 0.39 | 0.00 | 0.10 | 0.00 |  |  |  |  |  |  |
| mod_N07 | 2.80 | 4.60 | 5.80 | 6.00 | 5.80 | 4.60 | 0.80 |  |  |  | x | x | x |
| mod_N08 | 0.26 | 0.77 | 1.66 | 4.72 | 6.00 | 4.09 | 1.91 |  |  |  |  |  |  |
| mod_N09 | 1.44 | 4.32 | 6.00 | 4.08 | 1.20 | 0.96 | 0.24 |  |  |  |  | x | x |
| mod_N10 | 0.91 | 2.61 | 4.04 | 5.35 | 6.00 | 4.96 | 1.70 |  |  | x |  |  |  |
| mod_N11 | 0.81 | 2.24 | 4.88 | 6.00 | 3.76 | 1.83 | 0.41 |  |  |  |  |  |  |
| mod_N12 | 0.82 | 2.45 | 3.68 | 4.64 | 6.00 | 6.00 | 3.14 |  |  |  |  |  |  |
| mod_N13 | 0.30 | 0.30 | 0.44 | 1.26 | 3.78 | 6.00 | 2.44 |  |  | x | x | x |  |
|  |  | reliability estimates of item compositions: | | | | Cronbach’s *α* | |  | .87 | .85 | .84 | .77 | .77 |
|  |  |  |  |  |  | McDonald’s *ω* | |  | .87 | .85 | .84 | .78 | .78 |
|  |  |  | convergent validities of item compositions with the BFI-2: | | | | |  | .95 | .90 | .86 | .76 | .72 |
| *BFI-2 items, Openness:* | | |  |  |  |  |  |  |  |  |  |  |  |
| BFI_O01 | 0.40 | 0.40 | 0.80 | 6.00 | 4.40 | 2.50 | 0.70 |  |  |  |  |  |  |
| BFI_O02 | 0.57 | 1.05 | 1.43 | 6.00 | 3.33 | 1.33 | 0.76 |  |  |  | x |  | x |
| BFI_O03 | 0.69 | 1.85 | 1.96 | 6.00 | 3.92 | 2.31 | 0.81 |  |  |  | x | x | x |
| BFI_O04 | 0.18 | 0.28 | 0.46 | 6.00 | 2.31 | 3.42 | 1.38 |  |  |  |  |  |  |
| BFI_O05 | 0.00 | 0.00 | 0.08 | 0.59 | 1.94 | 6.00 | 4.23 |  | x | x | x | x | x |
| BFI_O06 | 0.71 | 0.71 | 2.24 | 4.71 | 6.00 | 2.47 | 1.06 |  |  |  |  |  |  |
| BFI_O07 | 0.10 | 0.21 | 0.21 | 0.83 | 3.62 | 6.00 | 4.76 |  | x |  |  |  |  |
| BFI_O08 | 0.00 | 0.35 | 1.06 | 3.29 | 5.76 | 6.00 | 1.41 |  |  |  |  |  |  |
| BFI_O09 | 0.11 | 0.00 | 0.42 | 0.84 | 3.05 | 6.00 | 5.58 |  | x | x |  |  |  |
| BFI_O10 | 0.09 | 0.26 | 0.26 | 0.87 | 2.26 | 6.00 | 3.48 |  | x |  |  |  |  |
| BFI_O11 | 0.00 | 0.12 | 0.82 | 3.76 | 6.00 | 5.41 | 1.76 |  | x | x | x | x | x |
| BFI_O12 | 0.00 | 0.10 | 0.31 | 1.83 | 2.85 | 6.00 | 4.37 |  | x | x | x |  |  |
| *Modified items, Openness:* | | |  |  |  |  |  |  |  |  |  |  |  |
| mod_O01 | 1.18 | 5.41 | 4.94 | 6.00 | 3.06 | 1.53 | 0.94 |  |  |  |  | x | x |
| mod_O02 | 0.10 | 0.60 | 1.10 | 2.70 | 6.00 | 3.40 | 1.30 |  |  | x |  |  |  |
| mod_O03 | 0.46 | 2.12 | 4.62 | 6.00 | 2.95 | 1.85 | 0.09 |  |  |  |  |  |  |
| mod_O04 | 0.68 | 4.36 | 6.00 | 4.64 | 2.86 | 1.64 | 0.55 |  |  |  |  |  |  |
| mod_O05 | 0.23 | 0.79 | 2.83 | 6.00 | 4.87 | 1.81 | 0.68 |  |  |  |  |  |  |
| mod_O06 | 0.32 | 0.56 | 1.76 | 6.00 | 1.92 | 1.36 | 0.24 |  |  |  |  |  |  |
| mod_O07 | 0.19 | 0.47 | 1.97 | 2.44 | 6.00 | 4.69 | 2.63 |  |  |  |  |  |  |
| mod_O08 | 1.11 | 4.00 | 6.00 | 2.11 | 2.11 | 1.00 | 0.56 |  |  |  |  | x | x |
| mod_O09 | 2.40 | 5.47 | 6.00 | 3.60 | 2.27 | 0.27 | 0.27 |  |  |  |  |  |  |
| mod_O10 | 0.15 | 0.68 | 1.67 | 6.00 | 4.56 | 1.59 | 0.23 |  |  |  |  |  |  |
| mod_O11 | 0.00 | 0.64 | 1.29 | 3.96 | 6.00 | 3.43 | 0.96 |  |  | x | x | x |  |
| mod_O12 | 1.32 | 6.00 | 4.27 | 2.85 | 0.81 | 0.10 | 0.10 |  |  |  |  |  |  |
|  |  | reliability estimates of item compositions: | | | | Cronbach’s *α* | |  | .83 | .81 | .81 | .75 | .82 |
|  |  |  |  |  |  | McDonald’s *ω* | |  | .84 | .81 | .82 | .77 | .84 |
|  |  |  | convergent validities of item compositions with the BFI-2: | | | | |  | .85 | .84 | .93 | .88 | .87 |
|  |  |  |  |  |  |  |  |  |  |  |  |  |  |

*Note.* Faking scoring weights refer to the case that negatively-keyed items are recoded. The five item compositions correspond to the compositions of desirability trajectories as displayed in Figure 1 in the Main Text. Reliability estimates and convergent validities are based on data from the low-stakes (LS) condition. BFI-2 = *Big Five Inventory 2* (Danner et al., 2016, 2019).

## Correlations Between Low-Stakes and High-Stakes Substantive Trait Person Parameters

**Table S.II.2**

*Correlations of Substantive Trait Person Parameters Between the Low-Stakes and High-Stakes Condition*

|  | Faking ignored | Faking modeled | *z* | *p* |
| --- | --- | --- | --- | --- |
| *Composition of desirability trajectories 1:* | | |  |  |
| E | .432 | .454 | 2.59 | .005 |
| A | .553 | .571 | 1.33 | .091 |
| C | .456 | .534 | 5.57 | <.001 |
| ES | .425 | .513 | 6.55 | <.001 |
| O | .512 | .514 | 0.14 | .445 |
| *Composition of desirability trajectories 2:* | | |  |  |
| E | .430 | .439 | 1.87 | .031 |
| A | .561 | .599 | 5.63 | <.001 |
| C | .556 | .587 | 9.19 | <.001 |
| ES | .429 | .460 | 5.38 | <.001 |
| O | .545 | .575 | 4.77 | <.001 |
| *Composition of desirability trajectories 3:* | | |  |  |
| E | .453 | .470 | 4.96 | <.001 |
| A | .610 | .617 | 1.44 | .075 |
| C | .582 | .608 | 8.01 | <.001 |
| ES | .436 | .478 | 7.08 | <.001 |
| O | .612 | .648 | 2.53 | .006 |
| *Composition of desirability trajectories 4:* | | |  |  |
| E | .466 | .482 | 4.61 | <.001 |
| A | .649 | .647 | –0.77 | .781 |
| C | .573 | .588 | 5.03 | <.001 |
| ES | .461 | .471 | 2.84 | .002 |
| O | .757 | .762 | 2.71 | .003 |
| *Composition of desirability trajectories 5:* | | |  |  |
| E | .510 | .518 | 2.65 | .004 |
| A | .609 | .640 | 4.37 | <.001 |
| C | .550 | .546 | –1.16 | 0.877 |
| ES | .496 | .504 | 2.46 | .007 |
| O | .811 | .810 | –1.20 | .884 |
|  |  |  |  |  |

*Note. N* = 1070. Differences in correlations were tested for significance with a *z*-test for overlapping correlations from dependent groups following Steiger (1980). *p*-values are one-tailed. To the low-stakes (LS) condition data, a model ignoring faking was fitted, whereas a model ignoring faking and a model accounting for faking were fitted to the high-stakes (HS) condition data. The five compositions of desirability trajectories correspond to those displayed in Figure 1 in the Main Text. Models ignoring faking only included dimensions for substantive traits and extreme response style (ERS), whereas models accounting for faking also included a faking dimension. E = Extraversion; A = Agreeableness; C = Conscientiousness; ES = Emotional Stability; O = Openness.

## Effects of Modeling Faking as a Standard Normal Distribution

In the modeling approach of this article, faking is treated as a normally distributed latent variable. To identify the model, the latent mean as well as the latent variance need to be defined. Throughout this article, we fixed the latent faking mean to 0 and the latent faking variance to 1, such that test-takers’ faking scores reflect *z*-scores.

As noted in the Main Text, the values to which the mean and variance of a latent variable are fixed are arbitrary. That is, fixing the latent mean and variance of the faking variable to any other real numbers than 0 and 1, respectively, would yield an equivalent model. Hence, the origin of the latent faking variable is not meaningful in an absolute sense. This implies that, in the case of faking centered around 0, positive versus negative faking scores do not reflect socially desirable (“faking good”) versus socially *un*desirable responding (“faking bad”). Instead, they just reflect above- versus below-average faking levels. In the data from the empirical demonstration, this is illustrated by comparing test-takers with faking scores above 0 to test-takers with faking scores below 0 regarding their responses in the high-stakes (HS) versus low-stakes (LS) condition. We therefore exemplarily considered the model accounting for substantive traits, ERS, and faking fitted to data from the HS condition where all items had monotonically increasing desirability trajectories. On this set of items (i.e., items where higher response categories were monotonically associated with higher desirability), test-takers with estimated faking scores above 0, on average, shifted their responses by 1.03 scale points (*Mdn* = 0.90, *IQR* = [0.30, 1.67]) into the desirable direction from the LS to the HS condition. Similarly, test-takers with estimated faking scores below 0 exhibited an average response shift of 0.40 scale points (*Mdn* = 0.33, *IQR* = [0.07, 0.63]) into the desirable direction. Note that the majority of test-takers with negative faking scores also engaged in socially desirable as opposed to socially *un*desirable responding, which is conceivable given the hypothetical application context. This illustrates that the latent faking variable captures the relative faking degree of test-takers instead of a qualitative faking distinction. Thus, a faking score of 0 also does not indicate the absence of faking but simply reflects the average faking level in the analyzed dataset.

Based on the modeling of test-takers’ faking degree, the model performs adjustments of substantive trait scores. To illustrate these adjustments, we compared the mean substantive trait scores for test-takers with positive versus negative faking scores from the model accounting for faking (model from above) to the corresponding substantive trait scores from a model ignoring faking. As can be seen in Table S.II.3, substantive trait scores were adjusted downward for test-takers with positive faking scores and upward for test-takers with negative faking scores. However, similar to the logic of not interpreting test-takers’ faking scores in an absolute sense, the numerical adjustments in terms of “upward” versus “downward” corrections of substantive trait scores shall only be interpreted as model-implied effects concerning adjustments of the relative rank order of test-takers.

**Table S.II.3**

*Mean Estimated Substantive Trait Scores in Models Without Vs. With Faking for Test-Takers With Negative Vs. Positive Faking Scores*

|  | Model ignoring faking | Model accounting for faking |
| --- | --- | --- |
| *Test-takers with faking scores < 0:* | | |
| E | –0.61 (0.67) | –0.49 (0.81) |
| A | –0.22 (0.71) | 0.12 (0.75) |
| C | –0.44 (0.71) | –0.16 (0.82) |
| ES | –0.59 (0.67) | –0.37 (0.85) |
| O | –0.55 (0.72) | –0.24 (0.84) |
| *Test-takers with faking scores > 0:* | | |
| E | 0.45 (0.65) | 0.22 (0.69) |
| A | 0.02 (0.86) | –0.34 (0.78) |
| C | 0.23 (0.77) | –0.16 (0.79) |
| ES | 0.41 (0.68) | 0.07 (0.71) |
| O | 0.37 (0.67) | –0.01 (0.74) |
|  |  |  |

*Note. N* = 1070. The model ignoring faking only included dimensions for substantive traits and extreme response style (ERS), whereas the model accounting for faking also included a faking dimension. Both models are based on data from the high-stakes (HS) condition and the item composition with only monotonically increasing desirability trajectories. The faking scores used to split the sample in this table are from the model accounting for faking. Numbers in brackets are standard deviations. E = Extraversion; A = Agreeableness; C = Conscientiousness; ES = Emotional Stability; O = Openness.

# References

Cao, M., Drasgow, F., & Cho, S. (2014). Developing ideal intermediate personality items for the ideal point model. *Organizational Research Methods, 18*(2), 252–275. <https://doi.org/10.1177/1094428114555993>

Danner, D., Rammstedt, B., Bluemke, M., Lechner, C., Berres, S., Knopf, T., Soto, C., & John, O. P. (2016). Die deutsche Version des Big Five Inventory 2 (BFI-2) [The German version of the Big Five Inventory 2 (BFI-2)]. *Zusammenstellung sozialwissenschaftlicher Items und Skalen (ZIS)* [*Compilation of items and scales for the social sciences (ZIS)*]. <https://doi.org/10.6102/zis247>

Danner, D., Rammstedt, B., Bluemke, M., Lechner, C., Berres, S., Knopf, T., Soto, C. J., & John, O. P. (2019). Das Big Five Inventar 2: Validierung eines Persönlichkeitsinventars zur Erfassung von 5 Persönlichkeitsdomänen und 15 Facetten [The Big Five Inventory 2: Validation of a personality inventory for measuring 5 personality domains 15 facets]. *Diagnostica, 65*(3), 121–132. <https://doi.org/10.1026/0012-1924/a000218>

Höft, S. (2019). Ermittlung eines beruflichen Anforderungsprofils [Determination of a job requirement profile]. In *Grundlagen der HR-Diagnostik* [*Foundations of HR assessment*] (pp. 12–28). European University of Applied Sciences Hamburg.

McCrae, R. R., & Costa, P. T. (1987). Validation of the five-factor model of personality across instruments and observers. *Journal of Personality and Social Psychology, 52*(1), 81–90. <https://doi.org/10.1037/0022-3514.52.1.81>

Schuler, H. (2014). Arbeits- und Anforderungsanalyse [Work and requirement analysis]. In H. Schuler & U. P. Kanning (Eds.), *Lehrbuch der Personalpsychologie* [*Textbook of personnel psychology*] (pp. 61–98). Hogrefe.

Steiger, J. H. (1980). Tests for comparing elements of a correlation matrix. *Psychological Bulletin, 87*(2), 245–251. <https://doi.org/10.1037/0033-2909.87.2.245>
